# Supplementary material for: Biomimetic NIR-II fluorescent proteins created from chemogenic protein-seeking dyes for multicolor deep-tissue bioimaging
Source: Nat Commun. 2024 Apr 2;15:2845. doi: 10.1038/s41467-024-47063-4 (PMC10987503; doi:10.1038/s41467-024-47063-4)
Supplement: Supplementary file 1 — Supplementary Information [file 41467_2024_47063_MOESM1_ESM.pdf]

Supplementary Information

Biomimetic NIR-II fluorescent proteins created from chemogenic protein-seeking dyes for multicolor deep-tissue bioimaging

Xu et al.

a

b

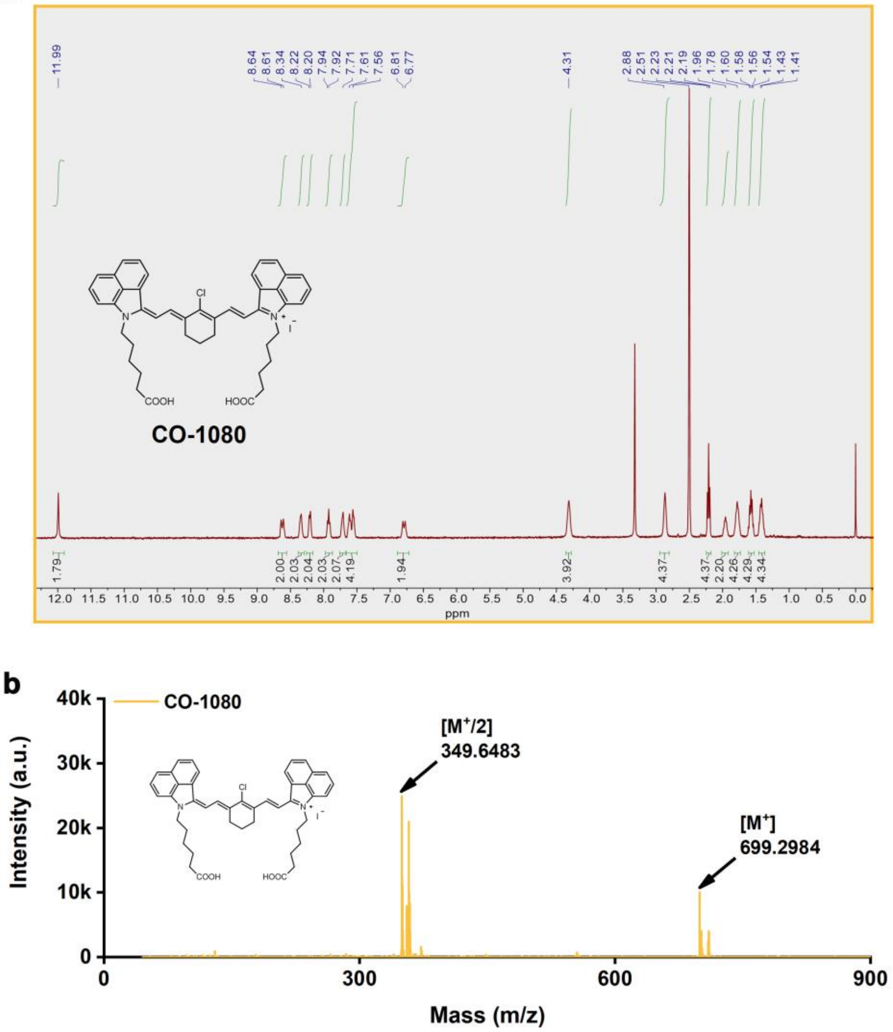

Supplementary Fig. 1. a ^{1}H-NMR spectra [^{1}H-NMR (400 MHz, DMSO-d_6) δ 11.99 (s, 2H), 8.62 (d, J = 13.2 Hz, 2H), 8.34 (d, J = 8.0 Hz, 2H), 8.21 (d, J = 7.6 Hz, 2H), 7.93 (t, J = 7.6 Hz, 2H), 7.71 (d, J = 8.0 Hz, 2H), 7.66 – 7.51 (m, 4H), 6.79 (d, J = 13.6 Hz, 2H), 4.38 – 4.26 (m, 4H), 2.95 – 2.81 (m, 4H), 2.21 (t, J = 7.2 Hz, 4H), 2.02 – 1.94 (m, 2H), 1.83 – 1.73 (m, 4H), 1.64 – 1.52 (m, 4H), 1.47 – 1.36 (m, 4H)] and **b** mass spectrometry of CO-1080 dye molecule. Source data are provided as a Source Data file.

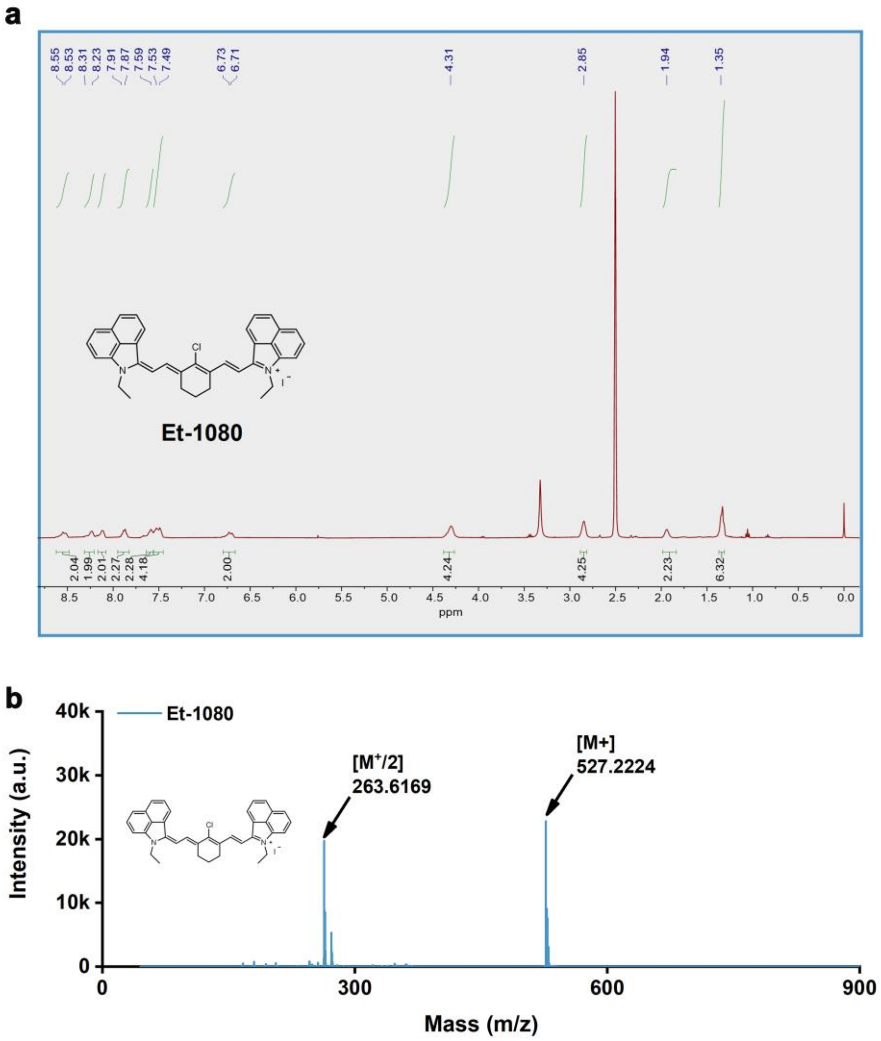

Supplementary Fig. 2. a ^{1}H-NMR spectra [^{1}H-NMR (400 MHz, DMSO-d_6) δ 8.54 (d, J = 14.0 Hz, 2H), 8.24 (d, J = 7.6 Hz, 2H), 8.12 (d, J = 8.0 Hz, 2H), 7.87 (t, J = 7.6 Hz, 2H), 7.60 (d, J = 8.0 Hz, 2H), 7.55 – 7.47 (m, 4H), 6.72 (d, J = 14.0 Hz, 2H), 4.30 (q, J = 7.2 Hz, 4H), 2.86 – 2.83 (m, 4H), 1.95 – 1.92 (m, 2H), 1.35 (t, J = 7.2 Hz, 6H)] and **b** mass spectrometry of Et-1080 dye molecule. Source data are provided as a Source Data file.

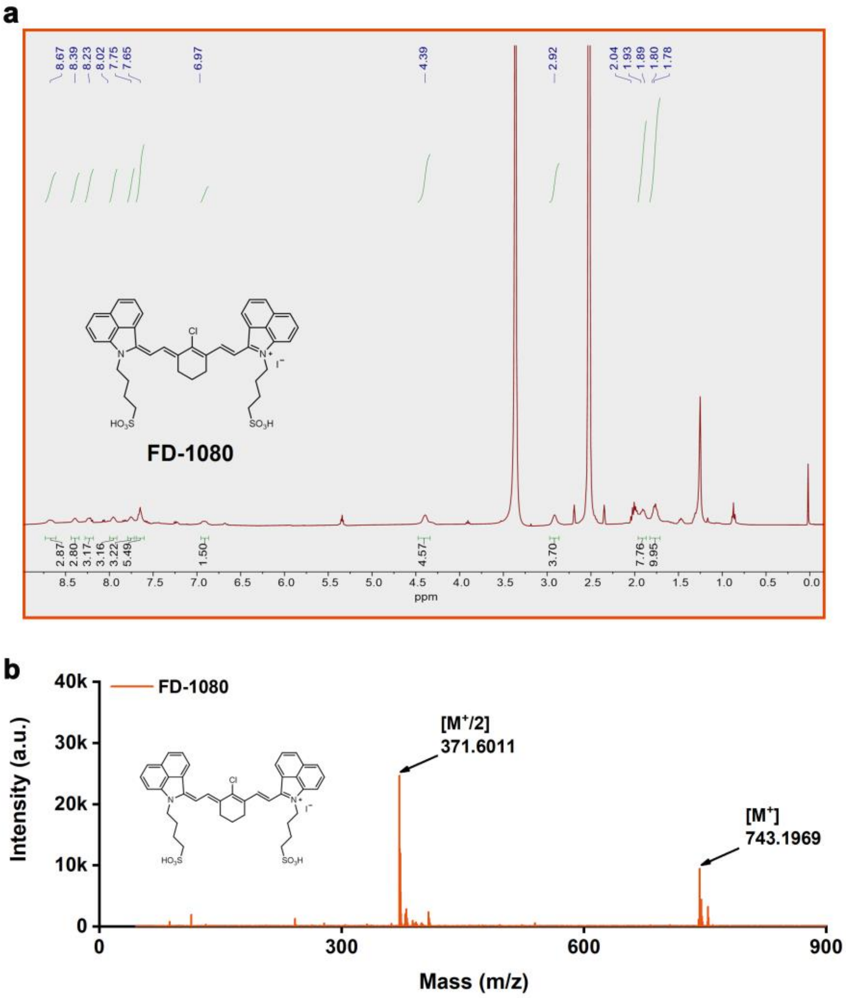

Supplementary Fig. 3. a ^{1}H-NMR spectra [^{1}H-NMR (400 MHz, DMSO-d_6) δ 8.72 – 8.61 (m, 2H), 8.47 – 8.30 (m, 2H), 8.26 – 8.14 (m, 2H), 8.02 – 7.89 (m, 2H), 7.81 – 7.69 (m, 2H), 7.68 – 7.55 (m, 4H), 6.98 – 6.80 (m, 2H), 4.45 – 4.32 (m, 4H), 2.96 – 2.80 (m, 4H), 1.95 – 1.83 (m, 6H), 1.80 – 1.65 (m, 8H)] and **b** mass spectrometry of FD-1080 dye molecule. Source data are provided as a Source Data file.

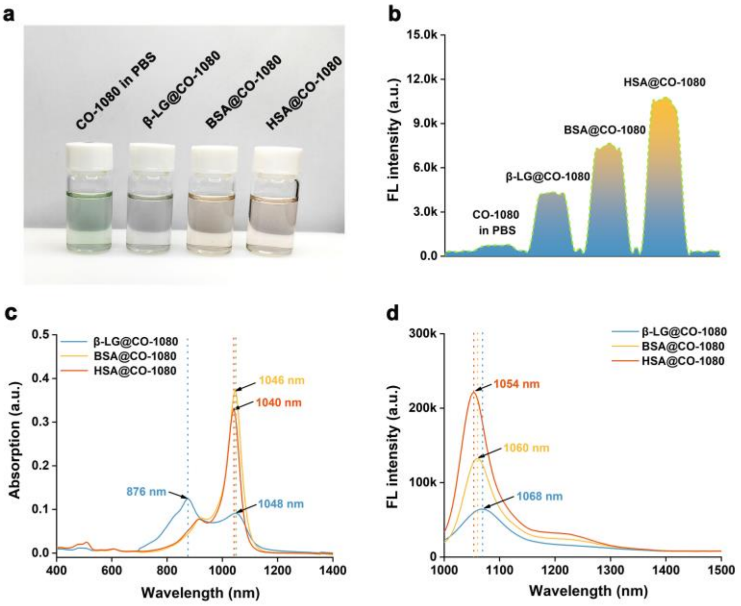

Supplementary Fig. 4. a Photographs of CO-1080, β-LG@CO-1080, BSA@CO-1080, and HSA@CO-1080 in PBS. **b** The fluorescence intensity CO-1080, β-LG@CO-1080, BSA@CO-1080, and HSA@CO-1080 in PBS. **c** UV-absorption spectroscopy and **d** fluorescence spectroscopy of the β-LG@CO-1080, BSA@CO-1080, and HSA@CO-1080. Source data are provided as a Source Data file.

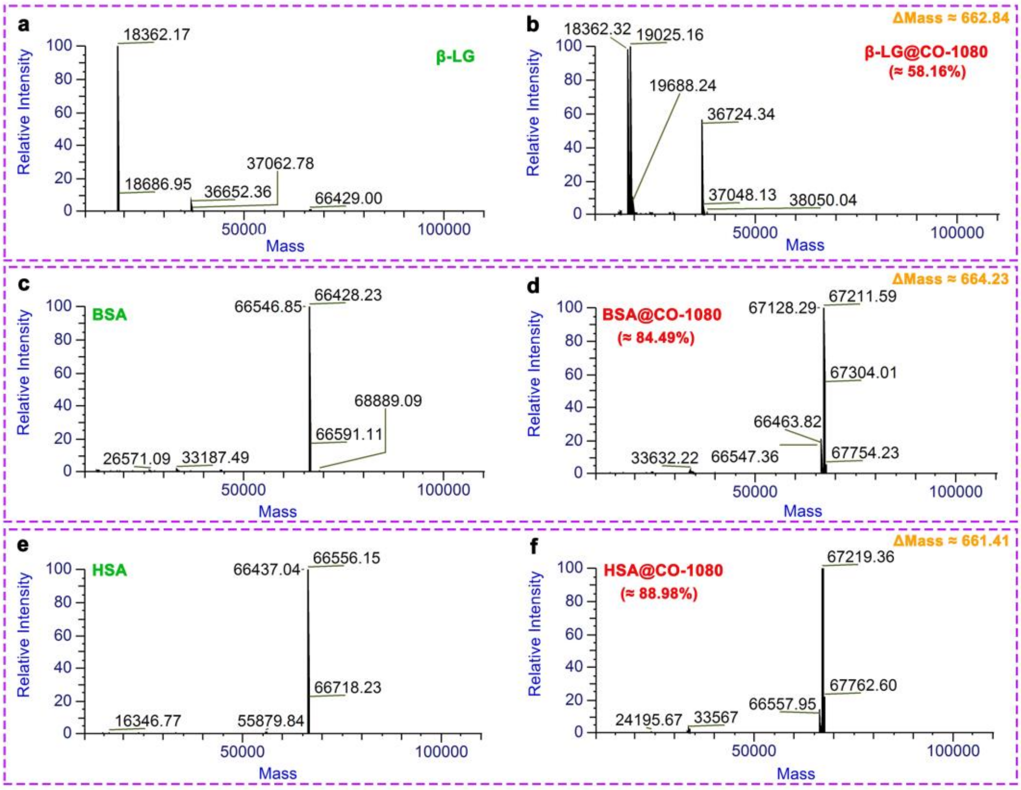

Supplementary Fig. 5. High-resolution mass spectrometry of **a** β-LG, **b** β-LG@CO-1080, **c** BSA, **d** BSA@CO-1080, **e** HSA, and **f** HSA@CO-1080.

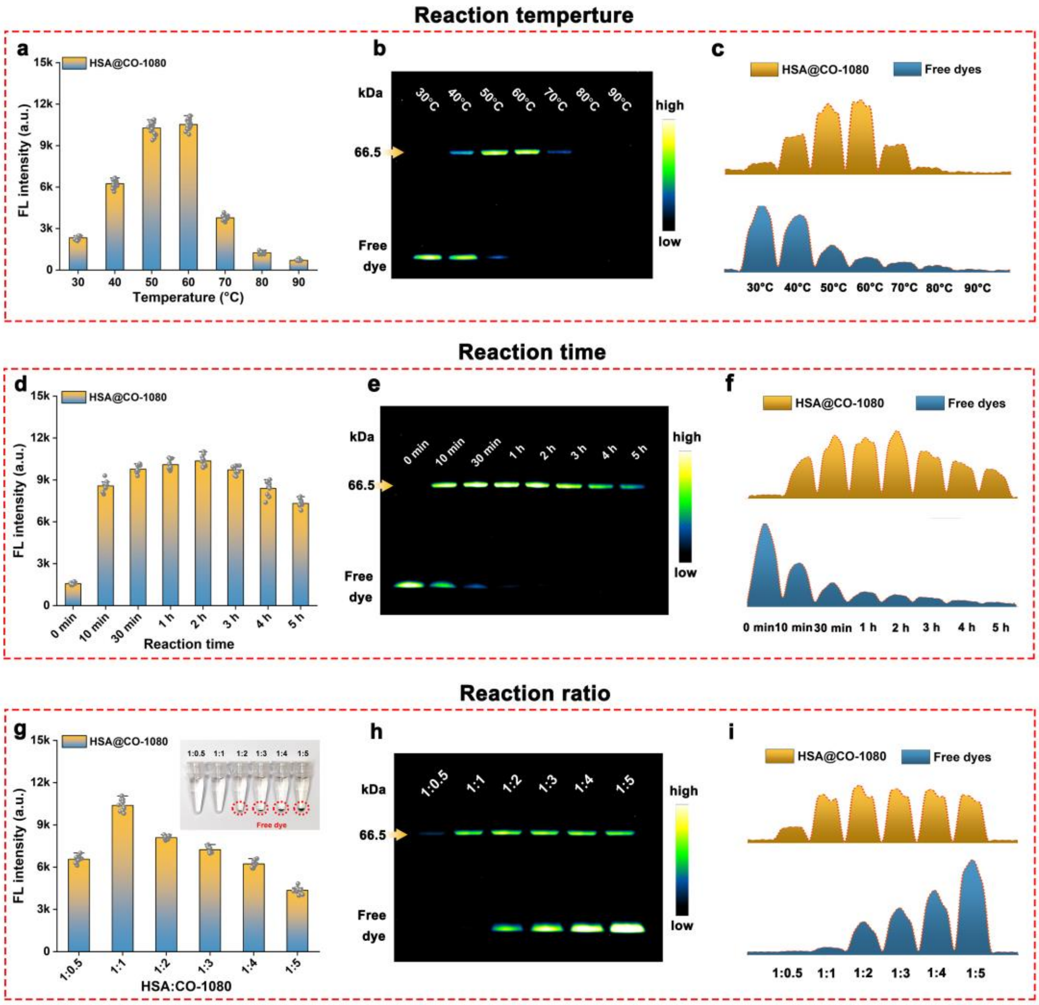

Supplementary Fig. 6. a NIR-II fluorescence intensity (mean ± SD, n = 10 independent samples per group), **b** electrophoresis analysis (n = 4 independent experiment), and **c** signal statistics of HSA@CO-1080 under different reaction temperatures. **d** NIR-II fluorescence intensity (mean ± SD, n = 10 independent samples per group), **e** electrophoresis analysis (n = 4 independent experiment), and **f** signal statistics of HSA@CO-1080 under different reaction time. **g** NIR-II fluorescence intensity (mean ± SD, n = 10 independent samples per group), **h** electrophoresis analysis (n = 4 independent experiment), and **i** signal statistics of HSA@CO-1080 under different reaction ratios. Source data are provided as a Source Data file.

Data note: i a reaction temperature of 60°C enabled sufficient covalent binding and fluorescence enhancement, while higher temperature caused denaturation of the HSA shell and greatly attenuated the NIR-II brightness; **ii** the optimal reaction time was 2 hours, as longer reaction time affected the stability of NIR-II FPs and led to luminescence degradation; and **iii** a reaction ratio between HSA and CO-1080 over 1:1 produced unbound free dye.

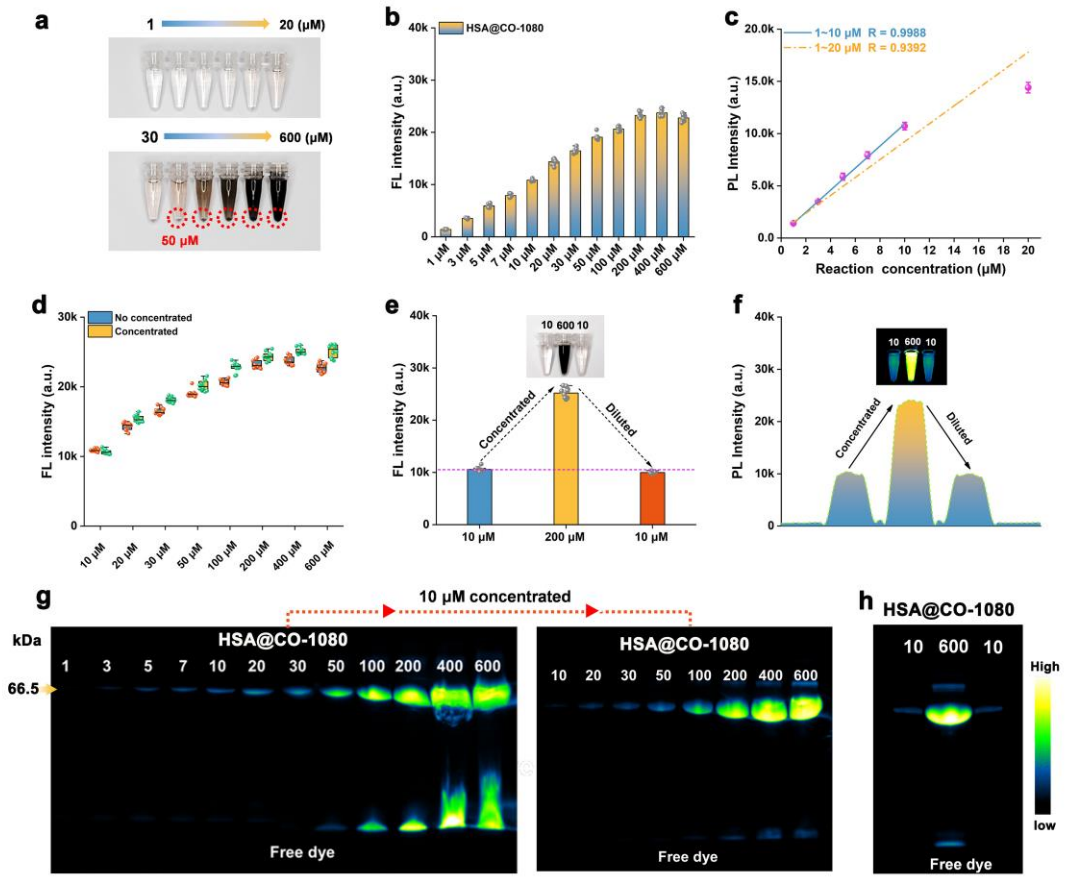

Supplementary Fig. 7. a Photograph and **b** fluorescence intensity of HSA@CO-1080 at different reaction concentrations (mean ± SD, n = 10 independent samples per group). **c** Linearly fitting of HSA@CO-1080 brightness against reaction concentrations. **d-f** Brightness comparison of the HSA@CO-1080 before/after concentration, followed by further dilution (mean ± SD, n = 10 independent samples per group). **g** Electrophoresis analysis of HSA@CO-1080 under different reaction concentrations (n = 4 independent experiment). **h** Electrophoresis analysis of HSA@CO-1080 before/after concentration, followed by further dilution (n = 4 independent experiment). Source data are provided as a Source Data file.

Data Note: As shown in Supplementary Fig. 7d-f, this approach effectively prevents intrinsic fluorescence quenching at high reaction concentrations, thereby improving the fluorescence intensity of the HSA@CO-1080 FPs. Notably, the NIR-II brightness of HSA@CO-1080 FPs completely recovers after diluting the concentrated sample back to its original concentration. The gel electrophoresis data also verified the effectiveness of the ultrafiltration concentration strategy to enhance CO-1080 dye utilization and amplify the application potential of HSA@CO-1080 FPs in in vivo imaging (Supplementary Fig. 7g-h). Taken together, we established a specific preparation process for HSA@CO-1080 FPs as displayed in Supplementary Fig. 8a.

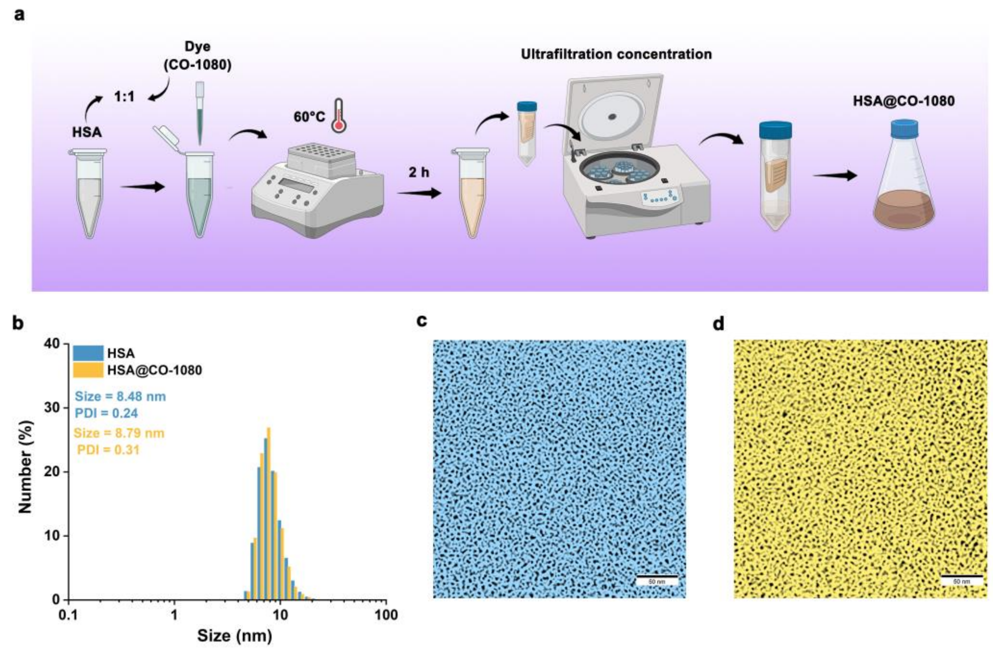

Supplementary Fig. 8. a Preparation flow chart of the HSA@CO-1080 probe. **b** Particle size of HSA and HSA@CO-1080 probe. Transmission electron microscopy (TEM) images of **c** HSA and **d** HSA@CO-1080 probe. Source data are provided as a Source Data file.

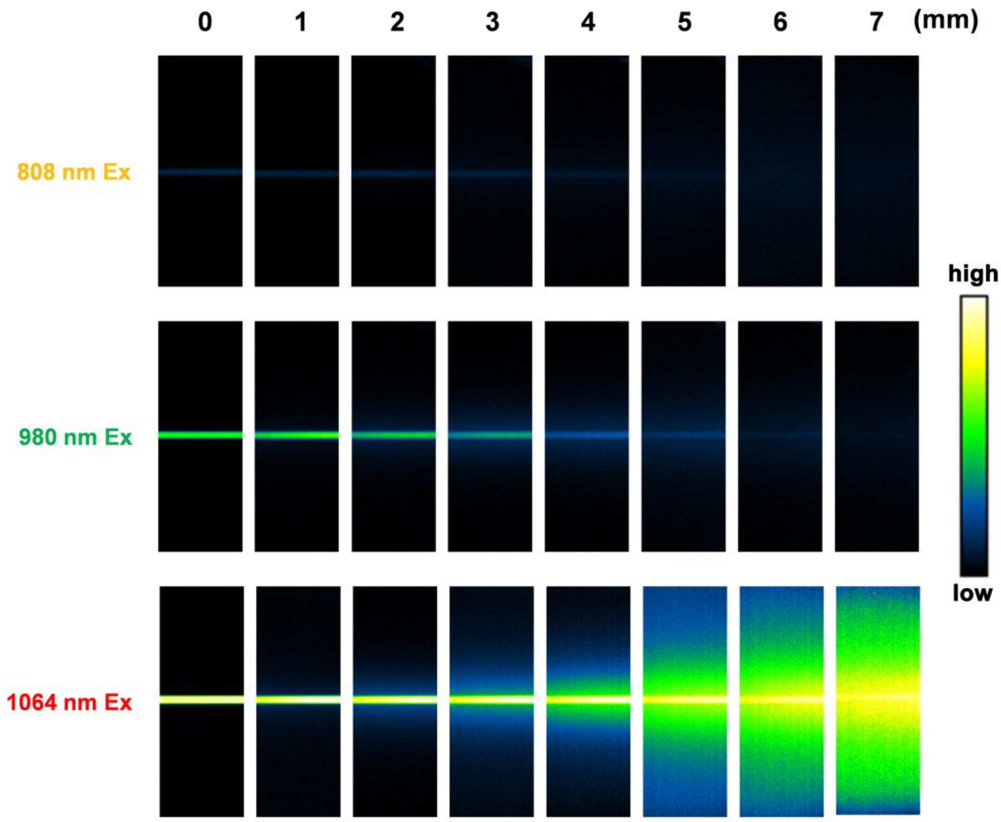

Supplementary Fig. 9. Comparison of the penetration depth of the HSA@CO-1080 under 808 nm, 980 nm, and 1064 nm laser excitation (the same exposure time).

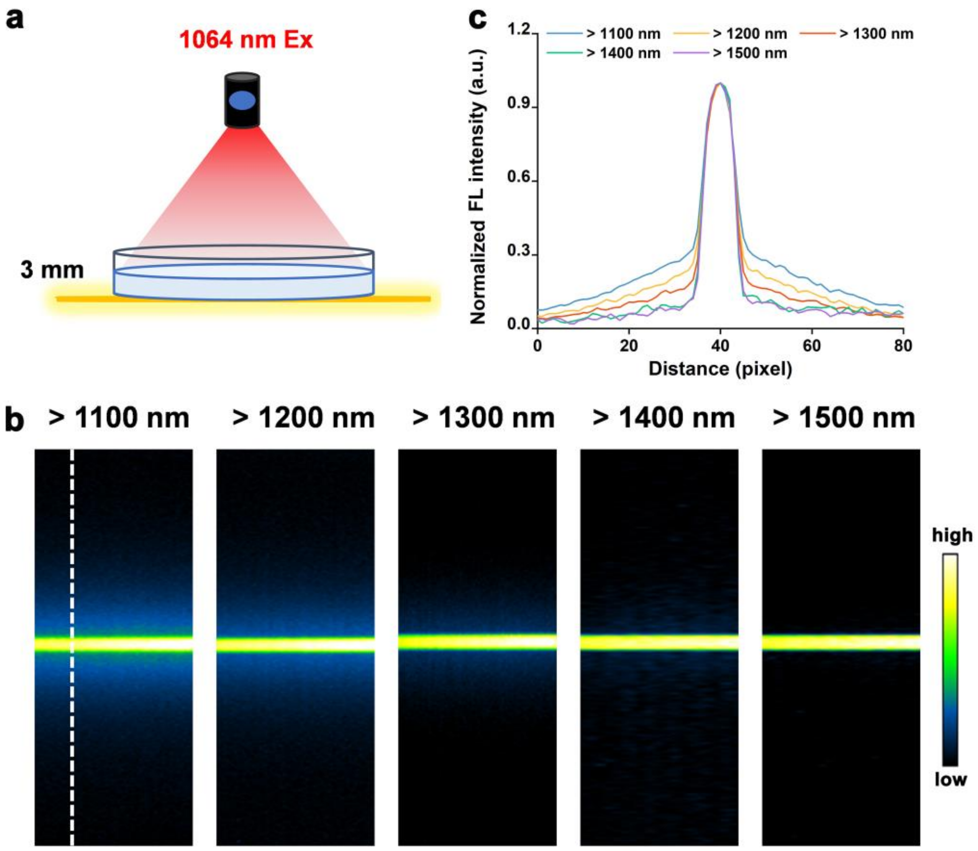

Supplementary Fig. 10. a Schematic of the HSA@CO-1080 penetration capability. **b-c** Comparison of the penetration ability of the HSA@CO-1080 at different imaging windows with 1064 nm laser excitation (normalized brightness under 3 mm thickness). Source data are provided as a Source Data file.

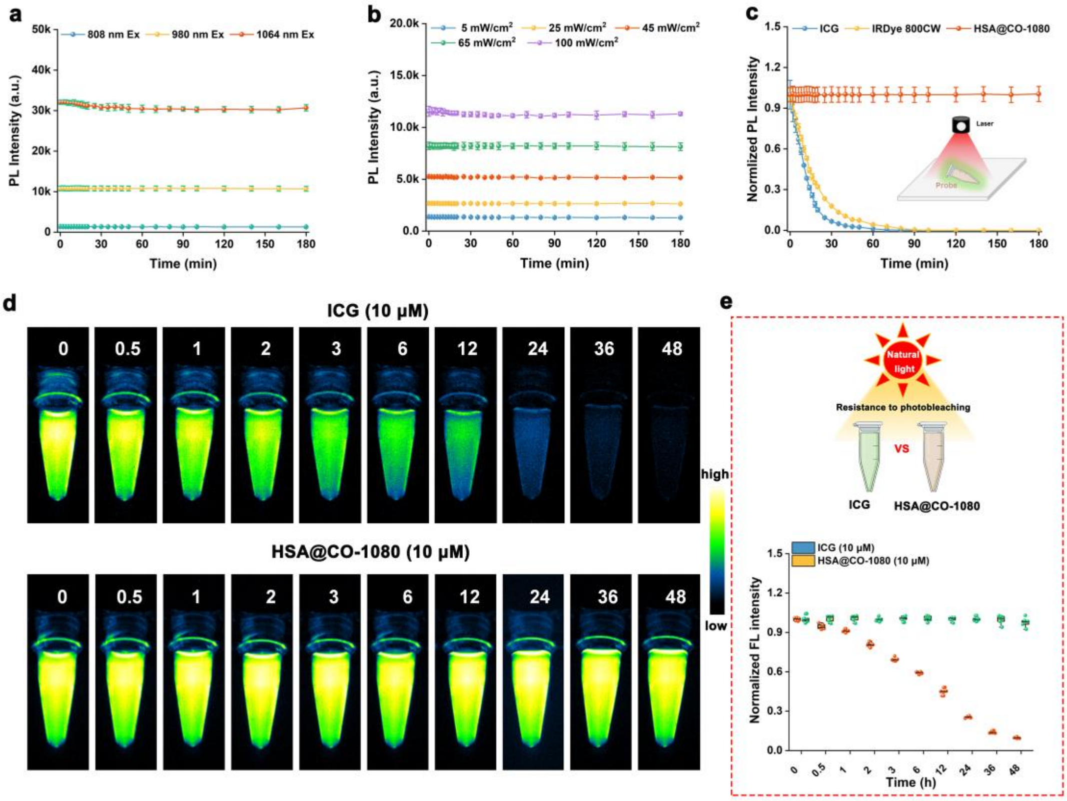

Supplementary Fig. 11. a Photostability of HSA@CO-1080 under 808 nm, 980 nm, and 1064 nm laser excitation (n = 3 independent samples per group). **b** Photostability of HSA@CO-1080 under different excitation power densities, including 5, 25, 45, 65, and 100 mW/cm^2 (n = 3 independent samples per group). **c** Comparison of the photostability of ICG, IRDye 800CW, and HSA@CO-1080 (n = 3 independent samples per group). **d-e** Comparison of photobleaching resistance between ICG and HSA@CO-1080 under natural light (n = 5 independent samples per group). Source data are provided as a Source Data file.

Data Note: The fluorescence intensity of the HSA@CO-1080 FPs remained almost unchanged under either continuous laser irradiation for 3 h or long-term inspection by excitation of different wavelengths (808 nm, 980 nm, and 1064 nm) and power densities (5~100 mW/cm^2) (Supplementary Fig. 11a-c). HSA@CO-1080 photobleached significantly less than ICG (Supplementary Fig. 11d, e). After 48 hours of natural light exposure, HSA@CO-1080 fluorescence properties remained almost unchanged while ICG showed significant fluorescence attenuation.

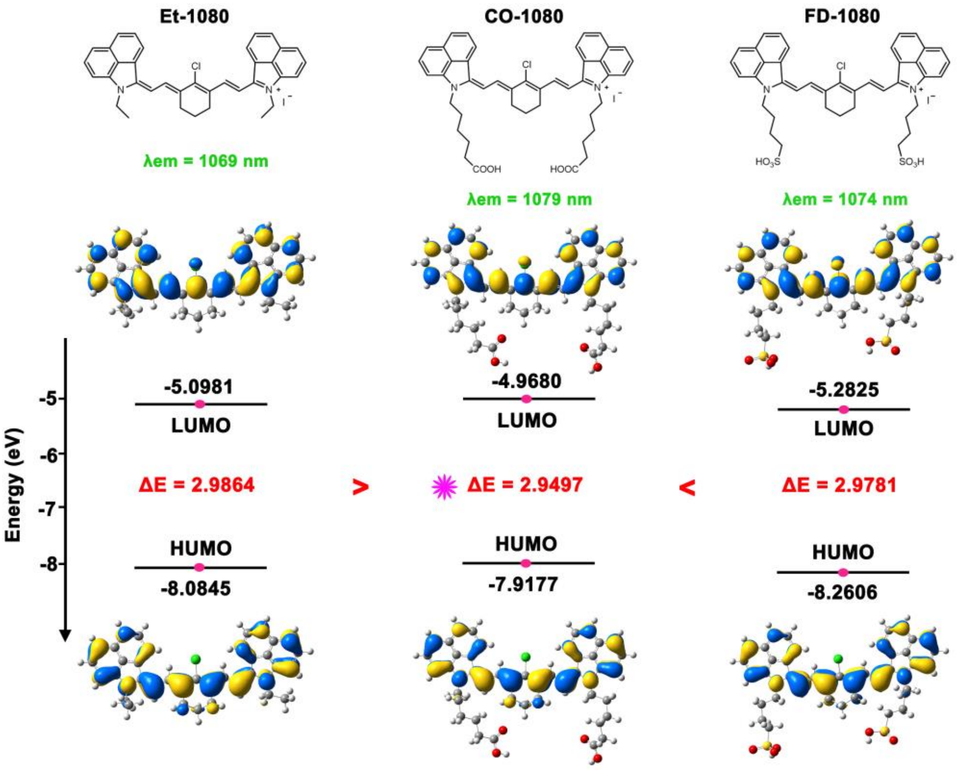

Supplementary Fig. 12. Schematic of HOMO and LUMO energy levels of the Et-1080, CO-1080, and FD-1080 dye calculated by density functional theory. The HOMO and LUMO energy levels were plotted based on the optimized S0 and S1 geometries using Gaussian (m062x/6-31 g (d, p)).

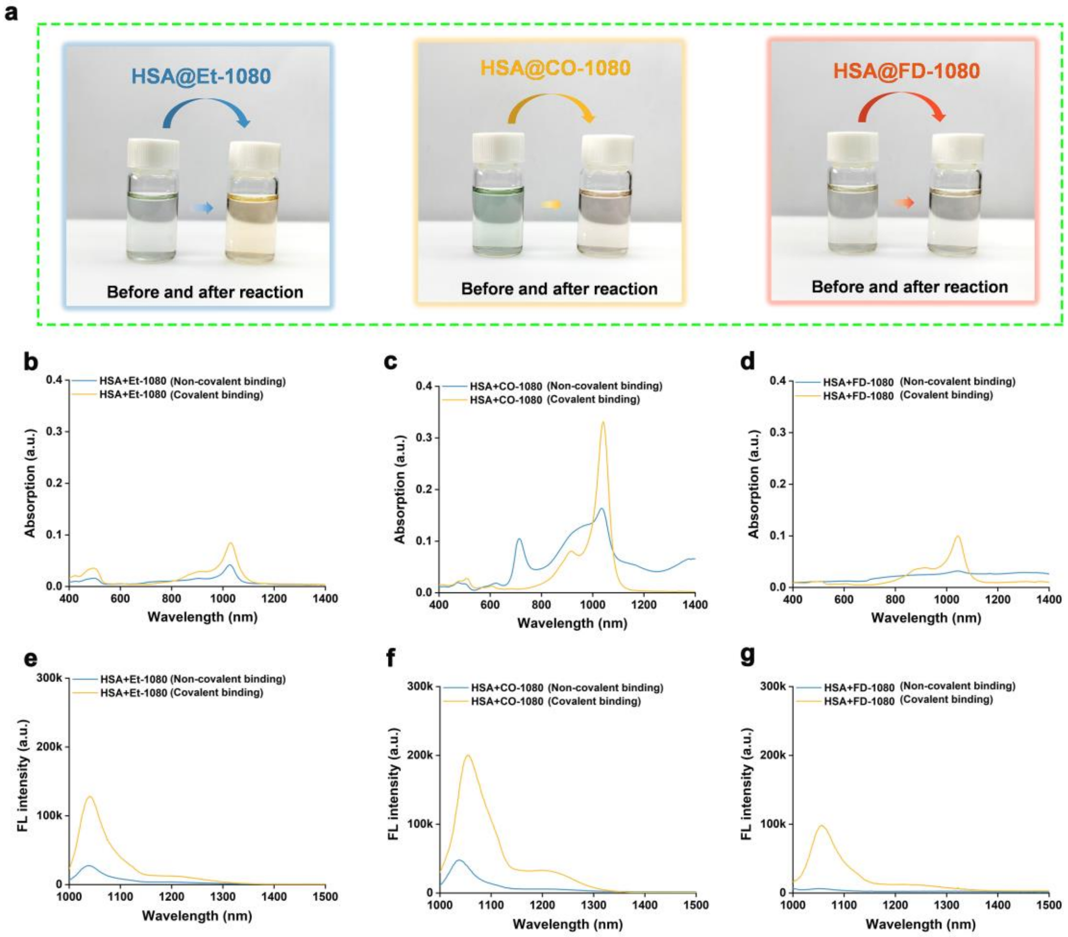

Supplementary Fig. 13. **a** Photographs of HSA@Et-1080, HSA@CO-1080, and HSA@FD-1080 before and after the reaction. **b** UV-absorption spectroscopy and **e** fluorescence spectroscopy of the HSA and Et-1080 before and after reaction. **c** UV-absorption spectroscopy and **f** fluorescence spectroscopy of the HSA and CO-1080 before and after reaction. **d** UV-absorption spectroscopy and **g** fluorescence spectroscopy of the HSA and Et-1080 before and after reaction. Source data are provided as a Source Data file.

Data Note: HSA and different dyes before the reaction (RT, direct mixing) were defined as non-covalent binding. HSA and different dyes after reaction (60°C, 2 h) were defined as covalent binding.

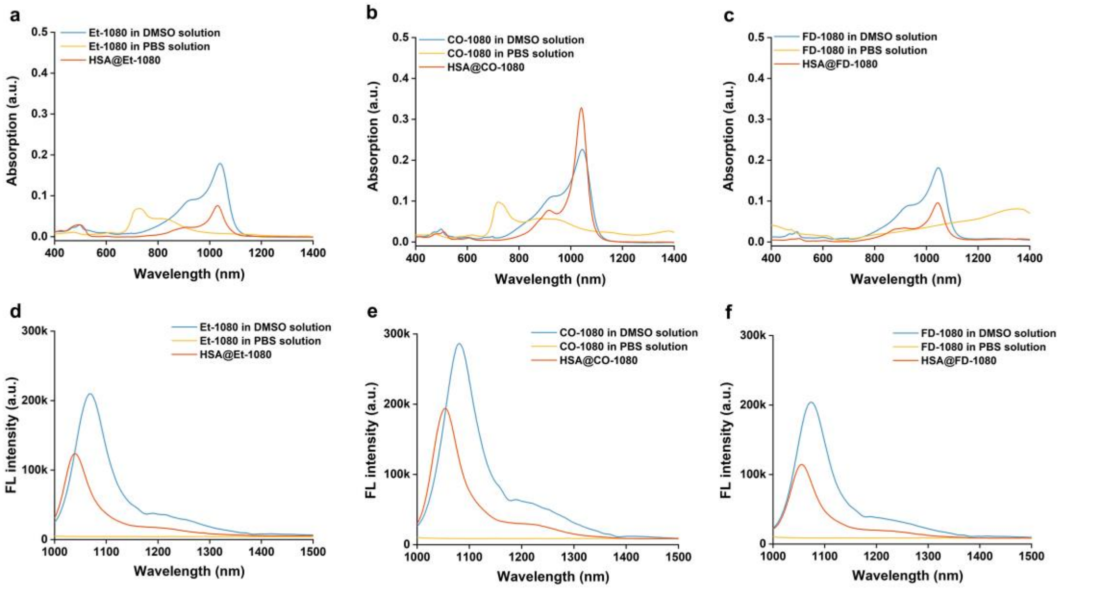

Supplementary Fig. 14. a UV-absorption spectra and **d** fluorescence spectra of Et-1080 in DMSO solution, Et-1080 in PBS solution, and HSA@Et-1080. **b** UV-absorption spectra and **e** fluorescence spectra of CO-1080 in DMSO solution, CO-1080 in PBS solution, and HSA@CO-1080. **c** UV-absorption spectra and **f** fluorescence spectra of FD-1080 in DMSO solution, FD-1080 in PBS solution, and HSA@FD-1080. Source data are provided as a Source Data file.

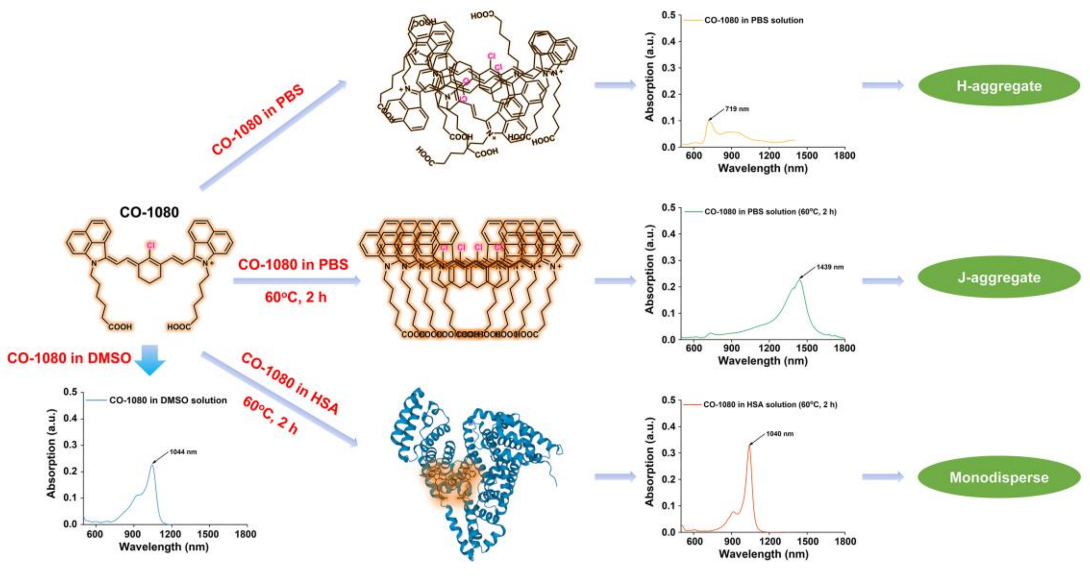

Supplementary Fig. 15. Schematic of the structure and properties of CO-1080 dyes under different reaction conditions. Protein structures were generated by the Protein Data Bank (PDB). Source data are provided as a Source Data file.

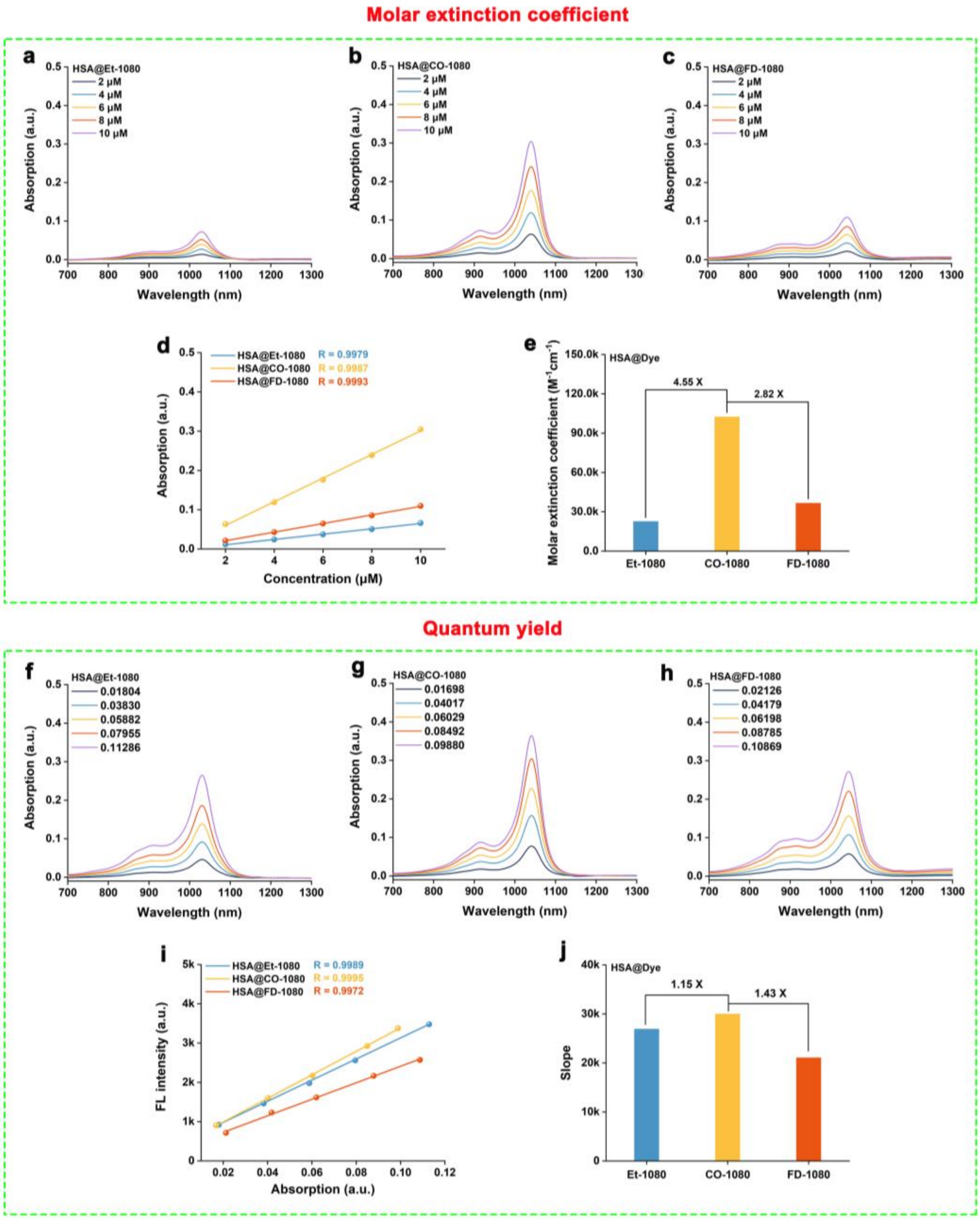

Supplementary Fig. 16. UV-absorption spectroscopy of **a** HSA@Et-1080, **b** HSA@CO-1080, and **c** HSA@FD-1080 at different gradient concentrations. **d-e** Comparison of molar extinction coefficients of HSA@Et-1080, HSA@CO-1080, and HSA@FD-1080. UV-absorption spectroscopy of **f** HSA@Et-1080, **g** HSA@CO-1080, and **h** HSA@FD-1080 at different gradient absorption. **i-j** Comparison of quantum yields of HSA@Et-1080, HSA@CO-1080, and HSA@FD-1080. Source data are provided as a Source Data file.

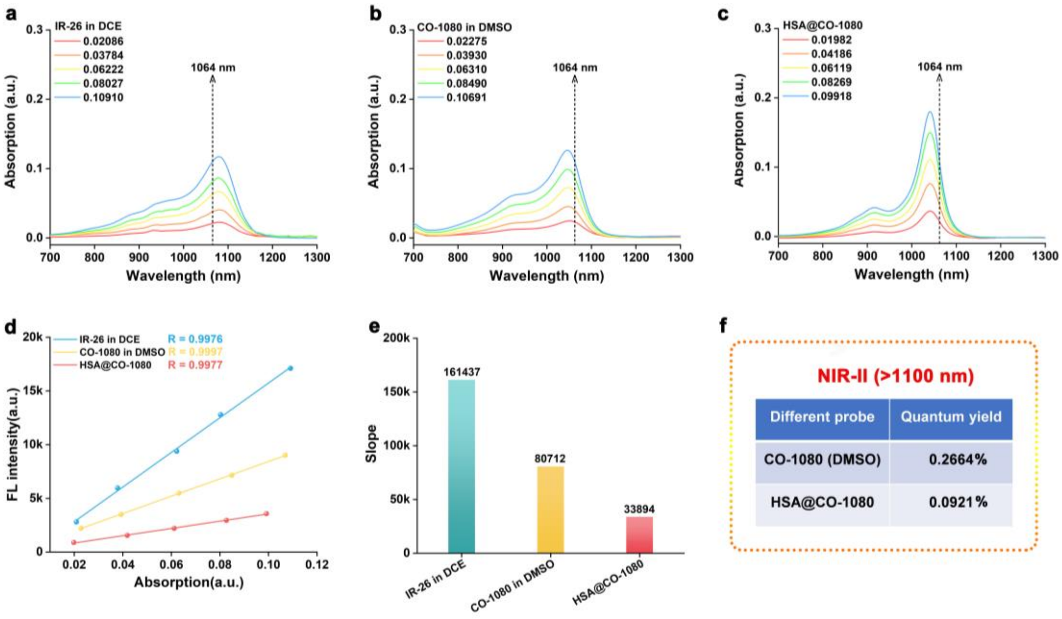

Supplementary Fig. 17. UV-absorption spectra of **a** IR-26 in dichloroethane (DCE), **b** CO-1080 in DMSO, and **c** HSA@CO-1080 at different gradient absorption. **d-e** Comparison of slope of IR-26 in dichloroethane (DCE), CO-1080 in DMSO, and HSA@CO-1080. **f** Quantum yields of CO-1080 in DMSO and HSA@CO-1080 (NIR-II, > 1100 nm). The quantum yield of IR-26 in DCE is 0.5% as a reference. Source data are provided as a Source Data file.

Supplementary Table 1. Summary of optical properties of NIR-II FPs

Dye	λ_{abs} (nm)	λ_{em} (nm)	Stokes shift (nm)	ε_{max} ($M^{-1}cm^{-1}$)	Slope	Brightness
HSA@Et-1080	1030	1039	9	22483	0.89 QY	20009.87 QY
HSA@CO-1080	1040	1054	14	102300	1.00 QY	102300.00 QY
HSA@FD-1080	1044	1055	11	36276	0.70 QY	25393.20 QY

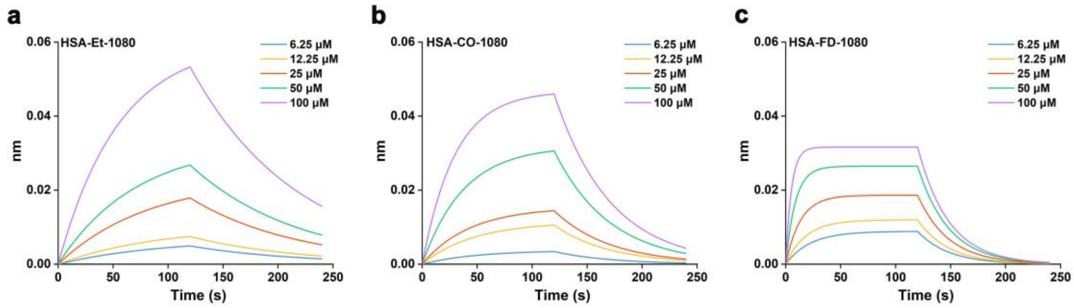

Supplementary Fig. 18. Kinetic binding assay of **a** Et-1080, **b** CO-1080, and **c** FD-1080 with HSA using bio-layer interferometry technique. Source data are provided as a Source Data file.

Supplementary Table 2. K_D, K_{on} and K_{off} for Et-1080, CO-1080, and FD-1080 with HSA

Protein	Dye	K_D (nM)	K_{on}	K_{off}
	Et-1080	14.5	1.04E+03	1.52E-02
HSA	CO-1080	2.13	1.09E+04	2.32E-02
	FD-1080	82.6	3.04E+03	2.51E-01

K_D: binding affinity; K_{on}: binding constant; K_{off}: dissociation constant

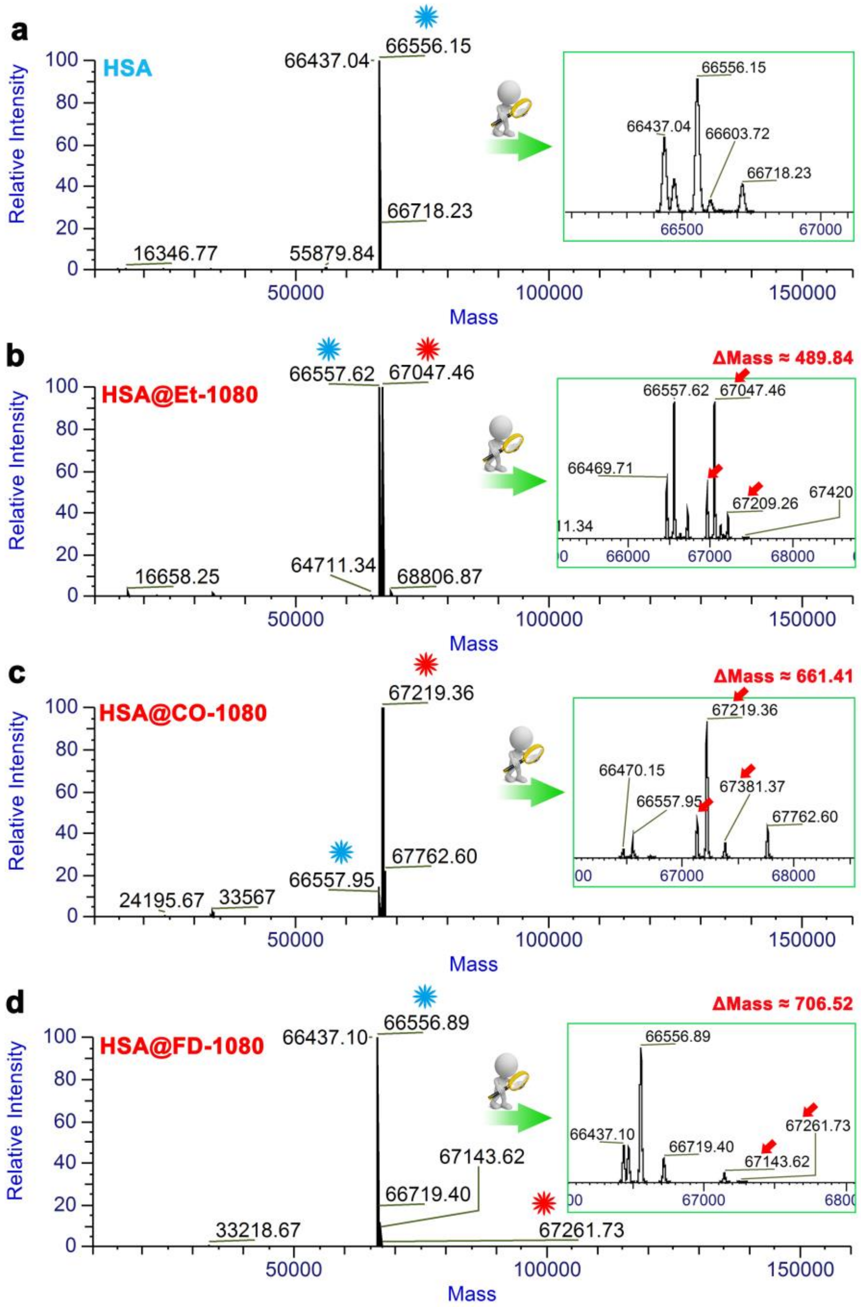

Supplementary Fig. 19. High-resolution mass spectrometry of **a** HSA, **b** HSA@Et-1080, **c** HSA@CO-1080, and **d** HSA@FD-1080.

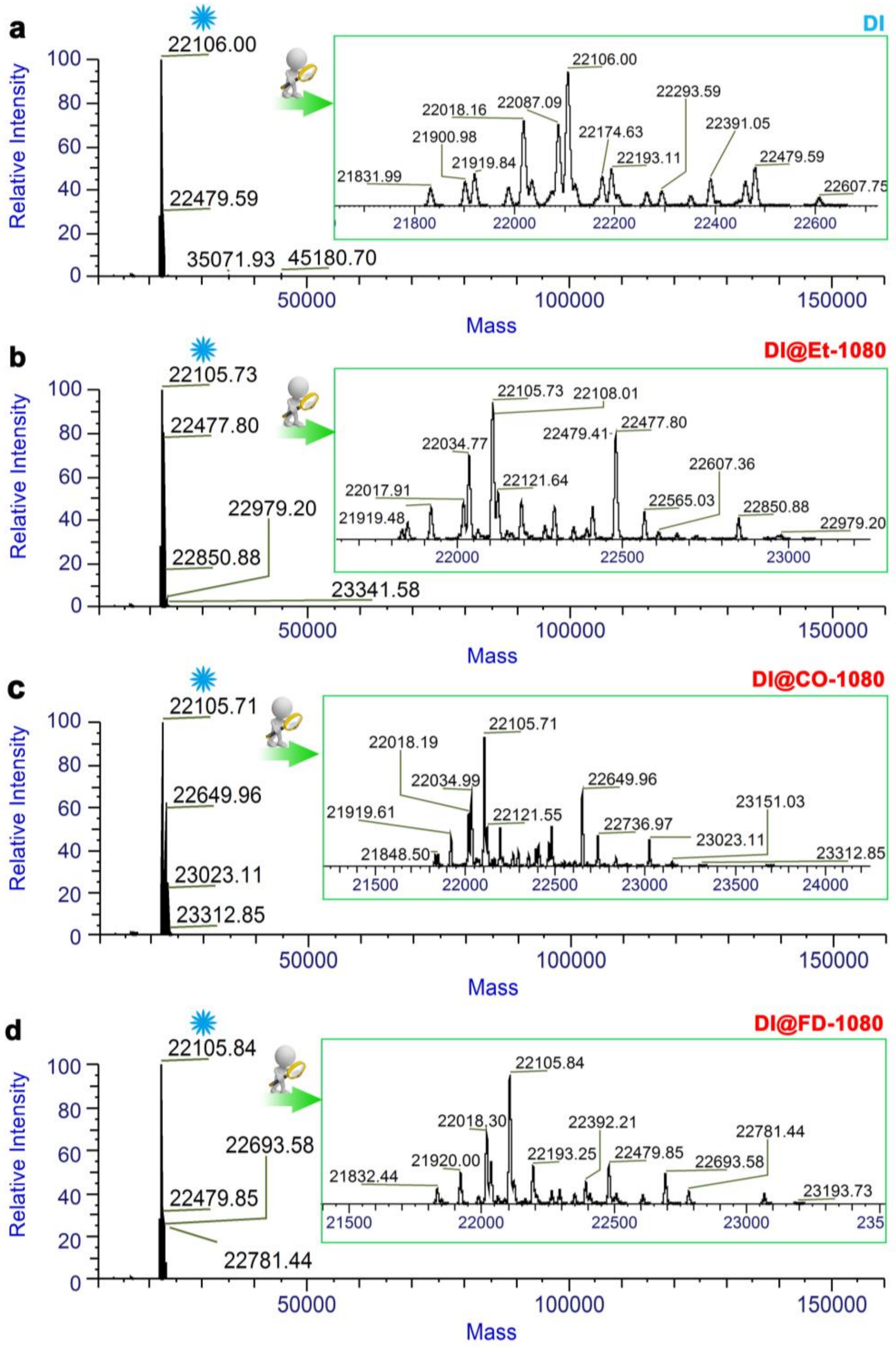

Supplementary Fig. 20. High-resolution mass spectrometry of **a** DI, **b** DI@Et-1080, **c** DI@CO-1080, and **d** DI@FD-1080.

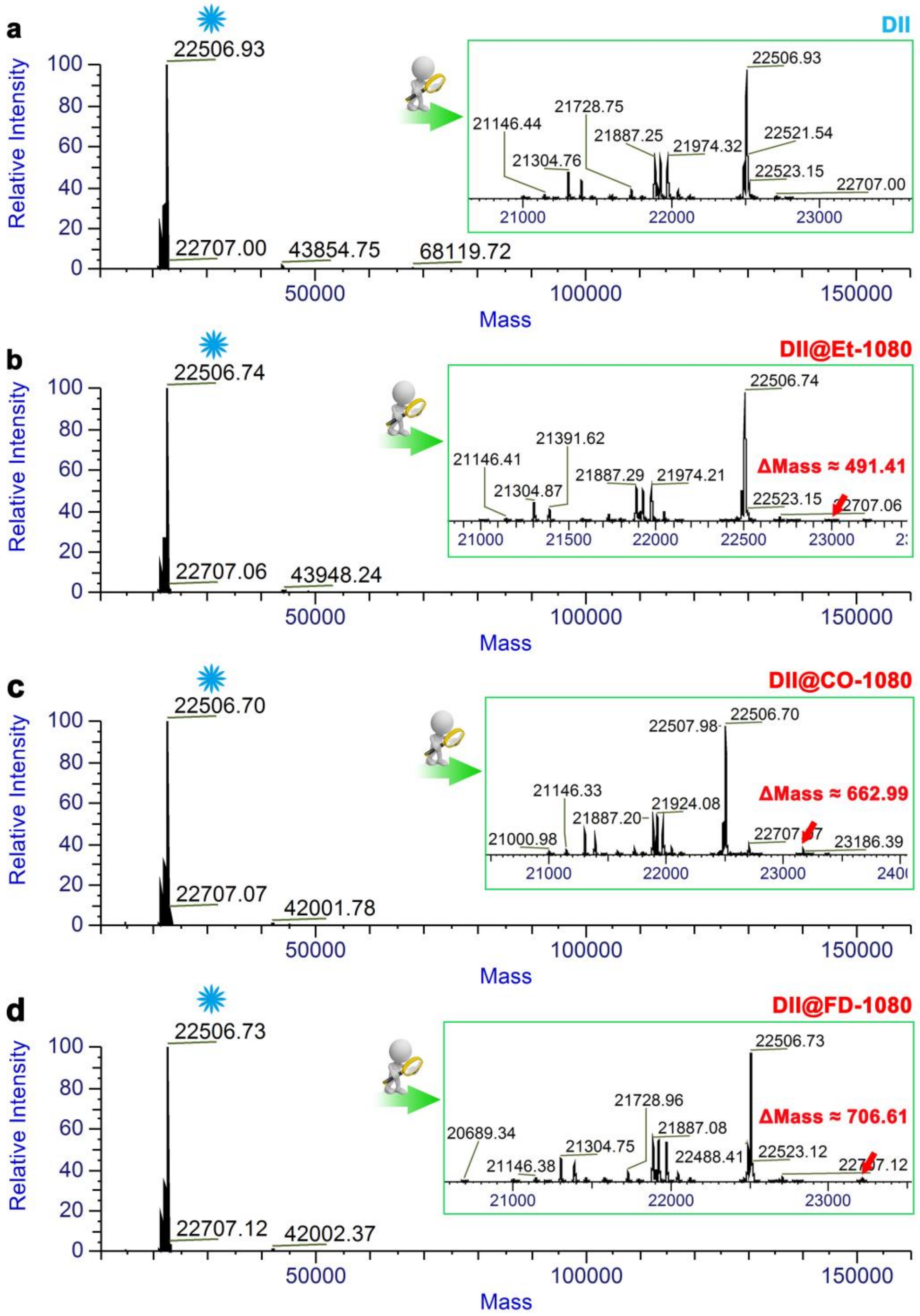

Supplementary Fig. 21. High-resolution mass spectrometry of **a** DII, **b** DII@Et-1080, **c** DII@CO-1080, and **d** DII@FD-1080.

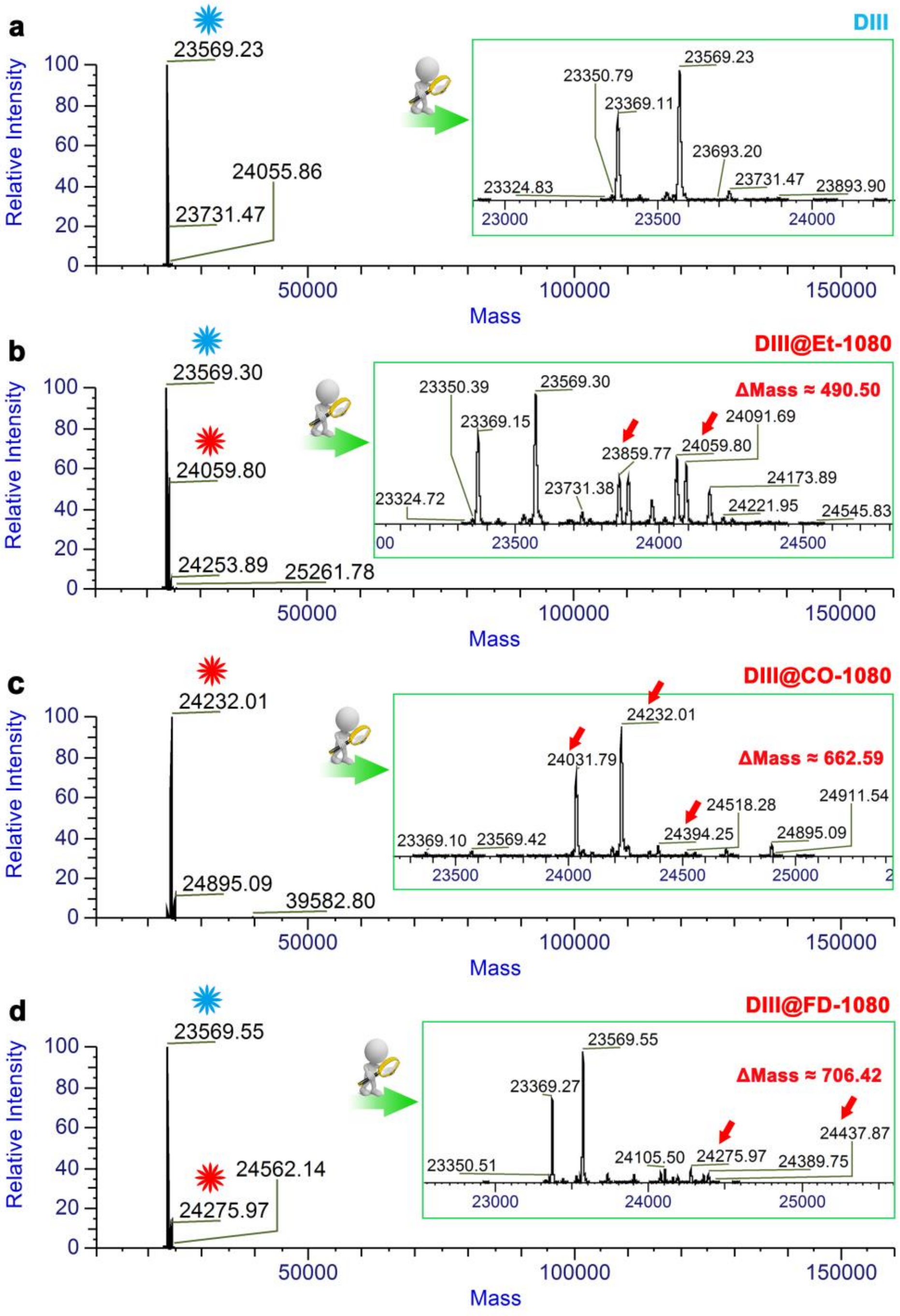

Supplementary Fig. 22. High-resolution mass spectrometry of **a** DIII, **b** DIII@Et-1080, **c** DIII@CO-1080, and **d** DIII@FD-1080.

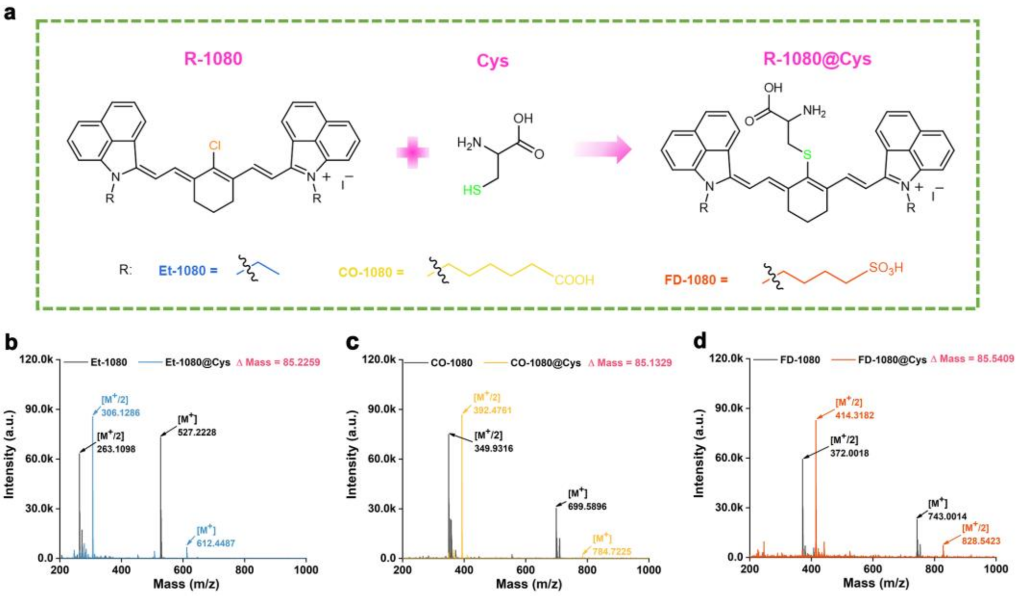

Supplementary Fig. 23. **a** Reaction diagram of R-1080 and cysteine (Cys). **b** The mass spectrometry of the Et-1080 and Et-1080@Cys. **c** The mass spectrometry of the CO-1080 and CO-1080@Cys. **d** The mass spectrometry of the FD-1080 and FD-1080@Cys. Source data are provided as a Source Data file.

Data note: It should be emphasized that the success of the above displacement reaction heavily relied on the base catalyst and an overdose of L-cysteine molecules (10:1 reaction ratio) addition.

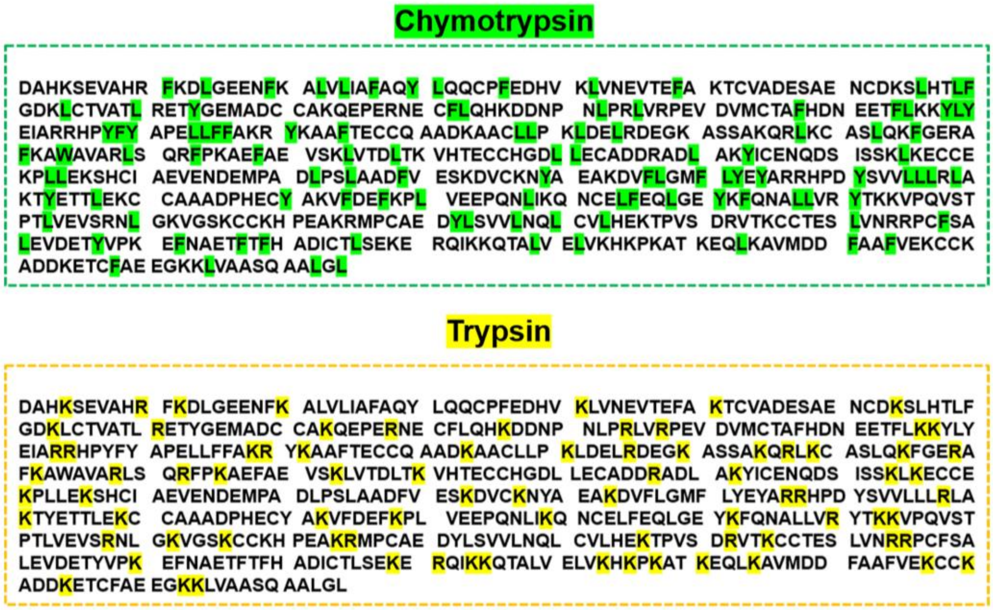

Supplementary Fig. 24. Digestion enzymes and their targeting cleavage sites on HSA.

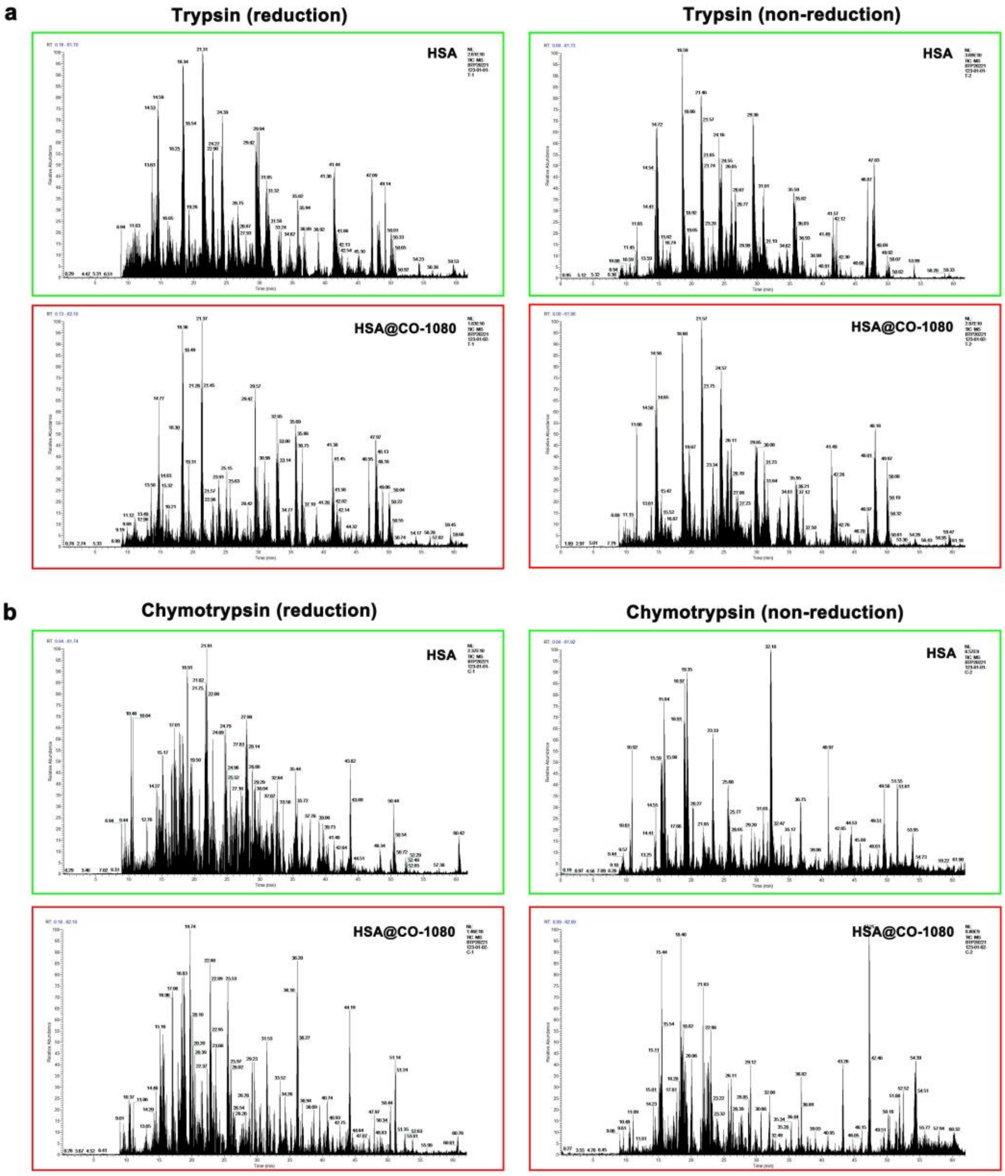

Supplementary Fig. 25. a Total ion flow chromatogram after trypsin enzyme digestion of HSA and HSA@CO-1080. **b** Total ion flow chromatogram after chymotrypsin enzyme digestion of HSA and HSA@CO-1080.

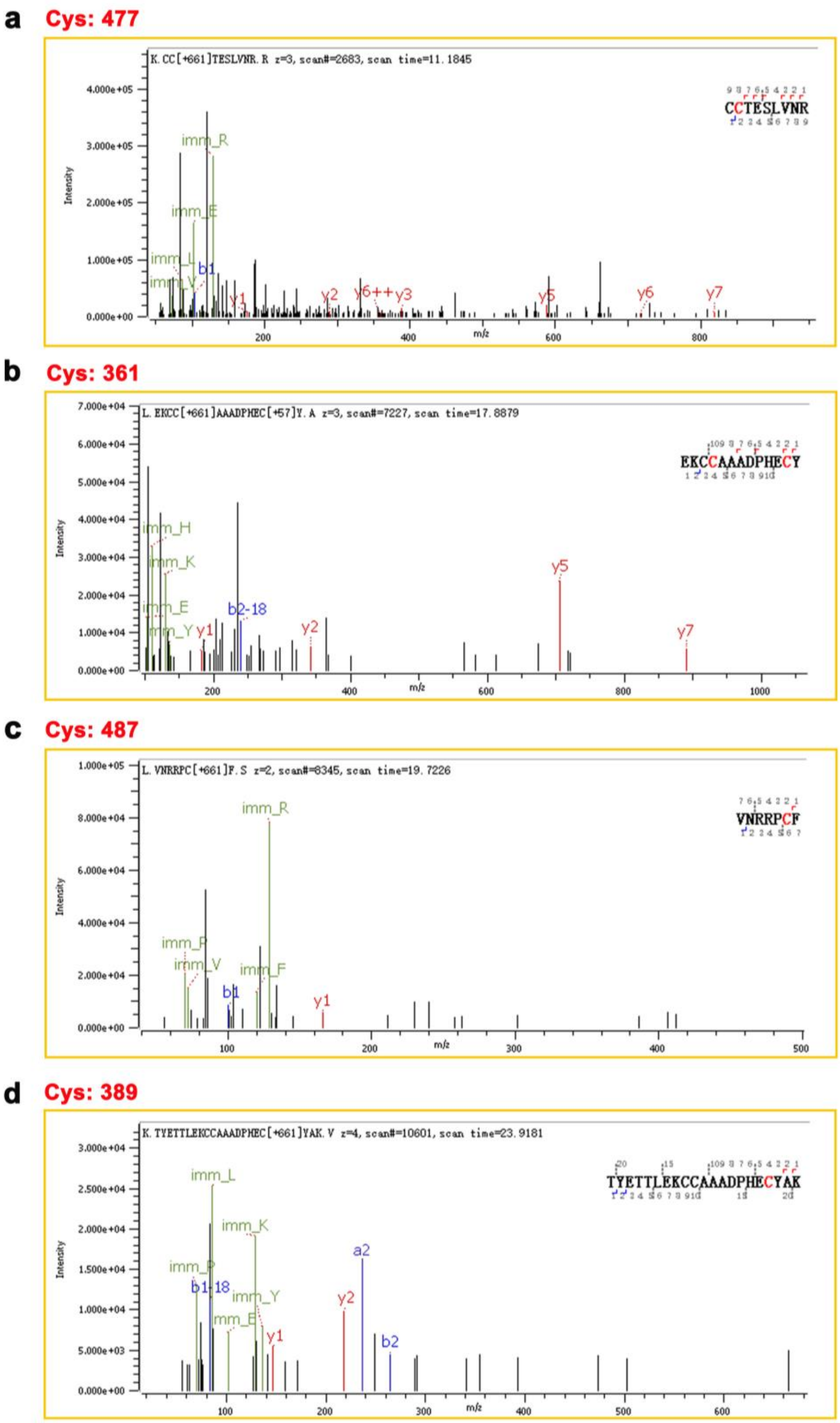

Supplementary Fig. 26. a The secondary mass spectra of the sequence of CCTESLVNR (Cys477). **b** The secondary mass spectra of the sequence of EKCCAAADPHECY (Cys361). **c** The secondary mass spectra of the sequence of VNRRPCF (Cys487). **d** The secondary mass spectra of the sequence of TYETTLEKCCAAADPHECYAK (Cys389).

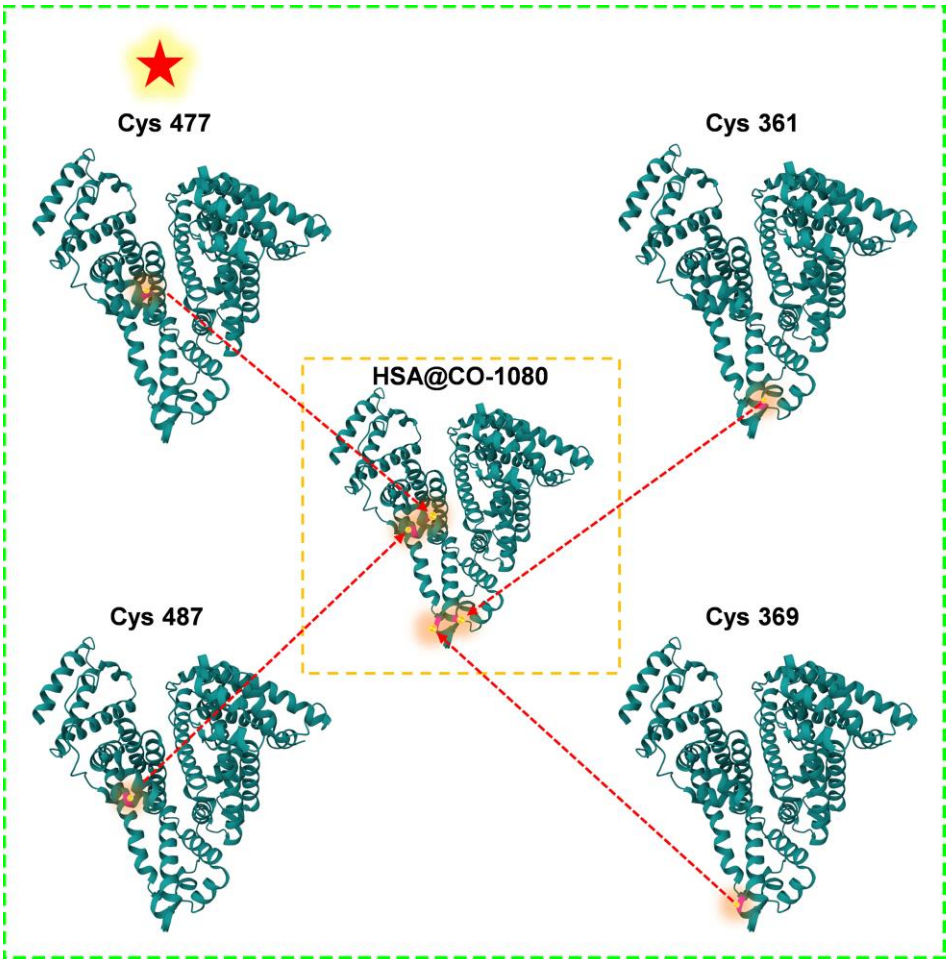

Supplementary Fig. 27. Distribution of cysteine sites recognized by proteomics. Protein structures were generated by the Protein Data Bank (PDB).

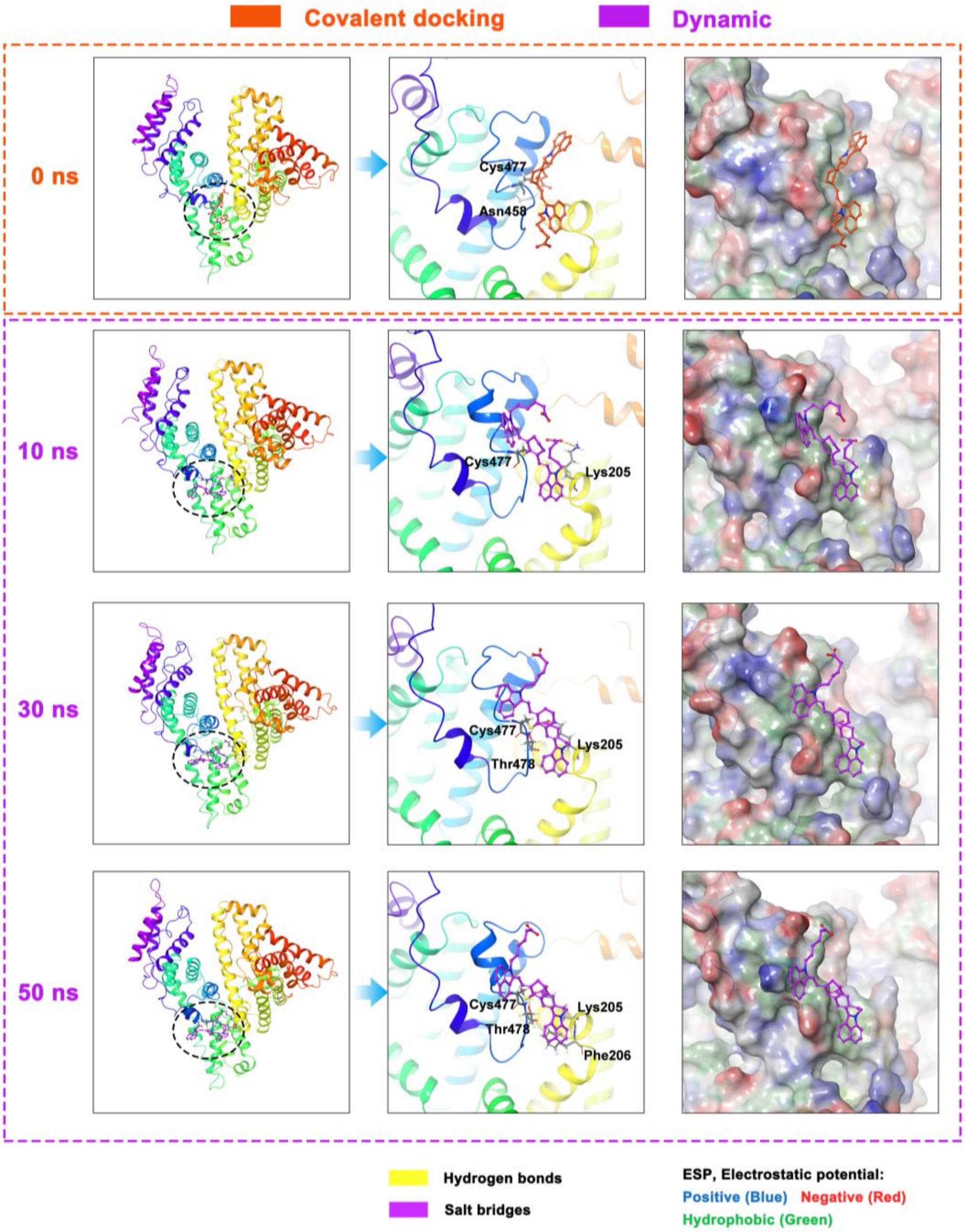

Supplementary Fig. 28. Schematic of protein-ligand interaction at different time points during the whole kinetic simulation process.

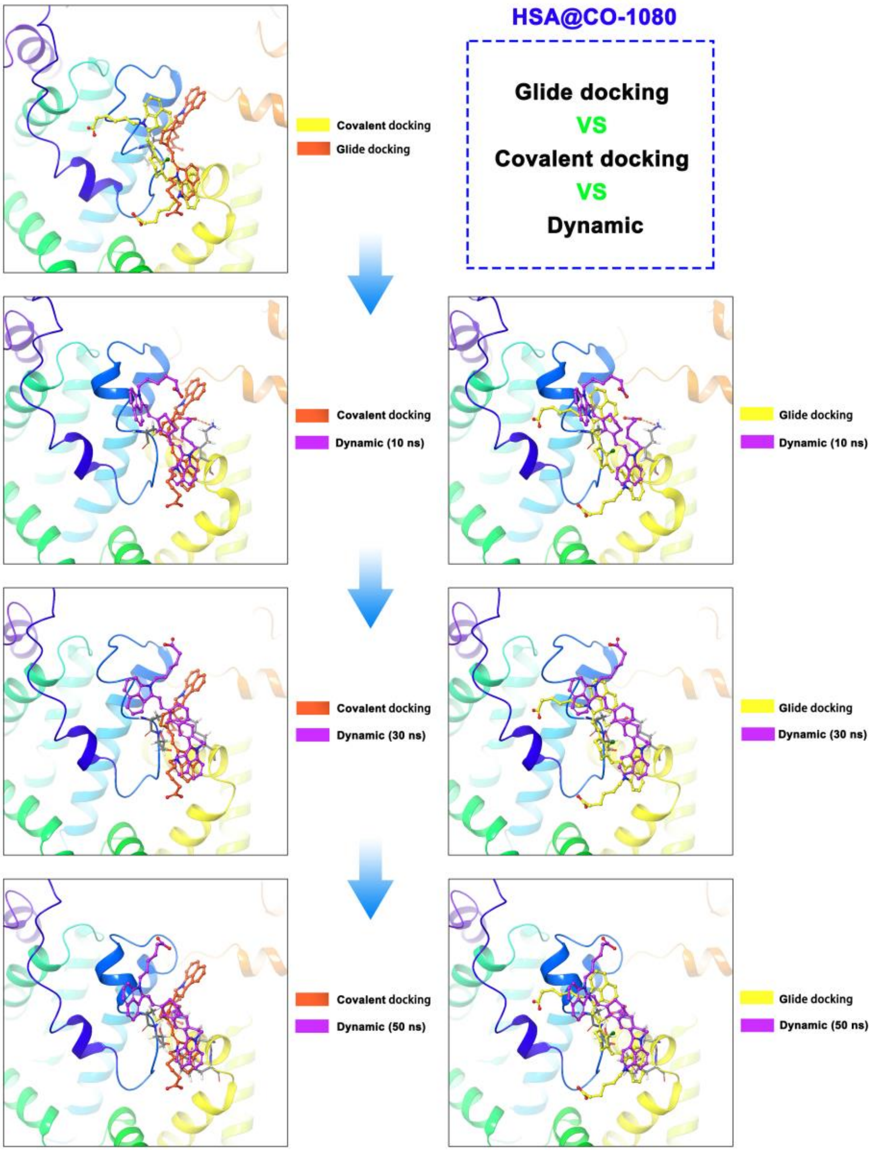

Supplementary Fig. 29. Comparison of the morphology of the ligand in the protein cavity at different time points in the kinetic simulation with that under glide and covalent docking simulations.

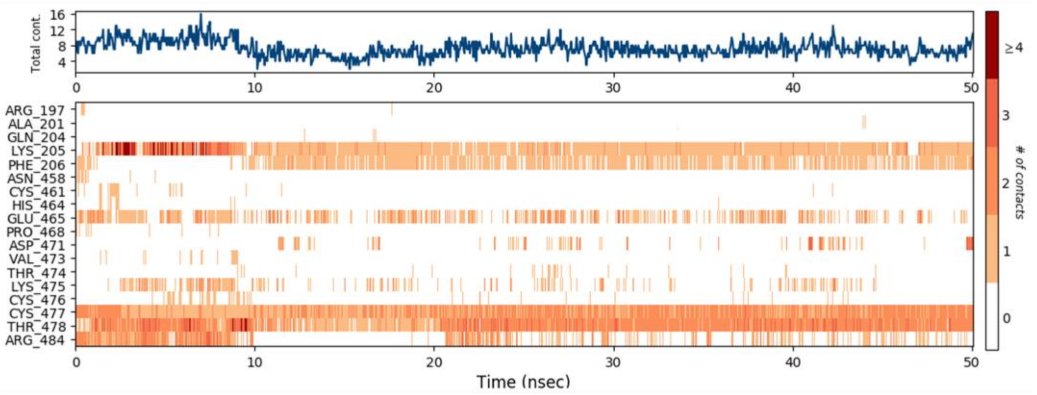

Supplementary Fig. 30. Protein-ligand interaction during the whole kinetic simulation.

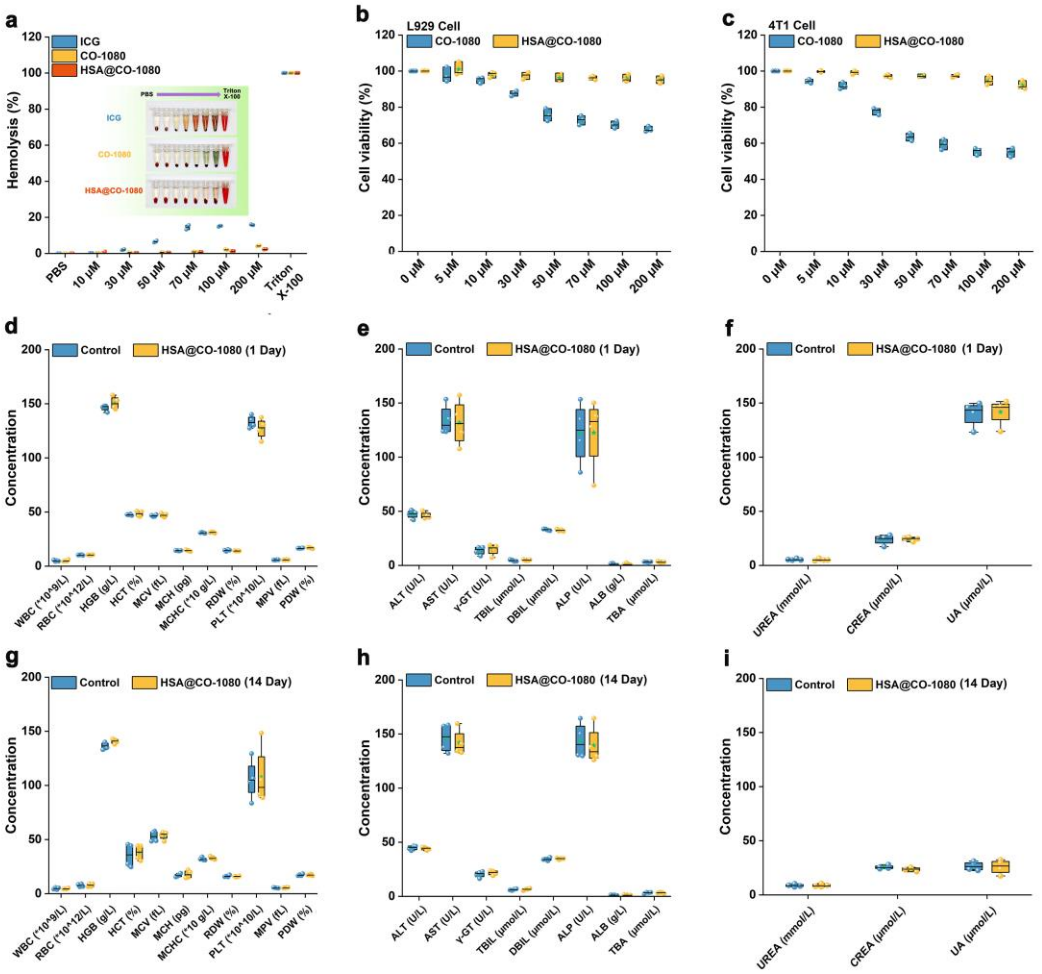

Supplementary Fig. 31. a Blood safety of ICG, CO-1080, and HSA@CO-1080 probes with different concentrations (n = 3 independent samples per group). Cytotoxicity of CO-1080 and HSA@CO-1080 probes with different concentrations on **b** the fibroblast cell (L929) and **c** the breast cancer cell (4T1) (n = 3 independent samples per group). **d** Blood routine indexes of mice after tail vein injection of the HSA@CO-1080 probes for 1 day (n = 4 independent samples per group). **e** Hepatic and **f** renal function indexes of mice after tail vein injection of the HSA@CO-1080 probes for 1 day (n = 4 independent samples per group). **g** Blood routine indexes of mice after tail vein injection of the HSA@CO-1080 probes for 14 days (n = 4 independent samples per group). **h** Hepatic and **i** renal function indexes of mice after tail vein injection of the HSA@CO-1080 probes for 14 days (n = 4 independent samples per group). Source data are provided as a Source Data file.

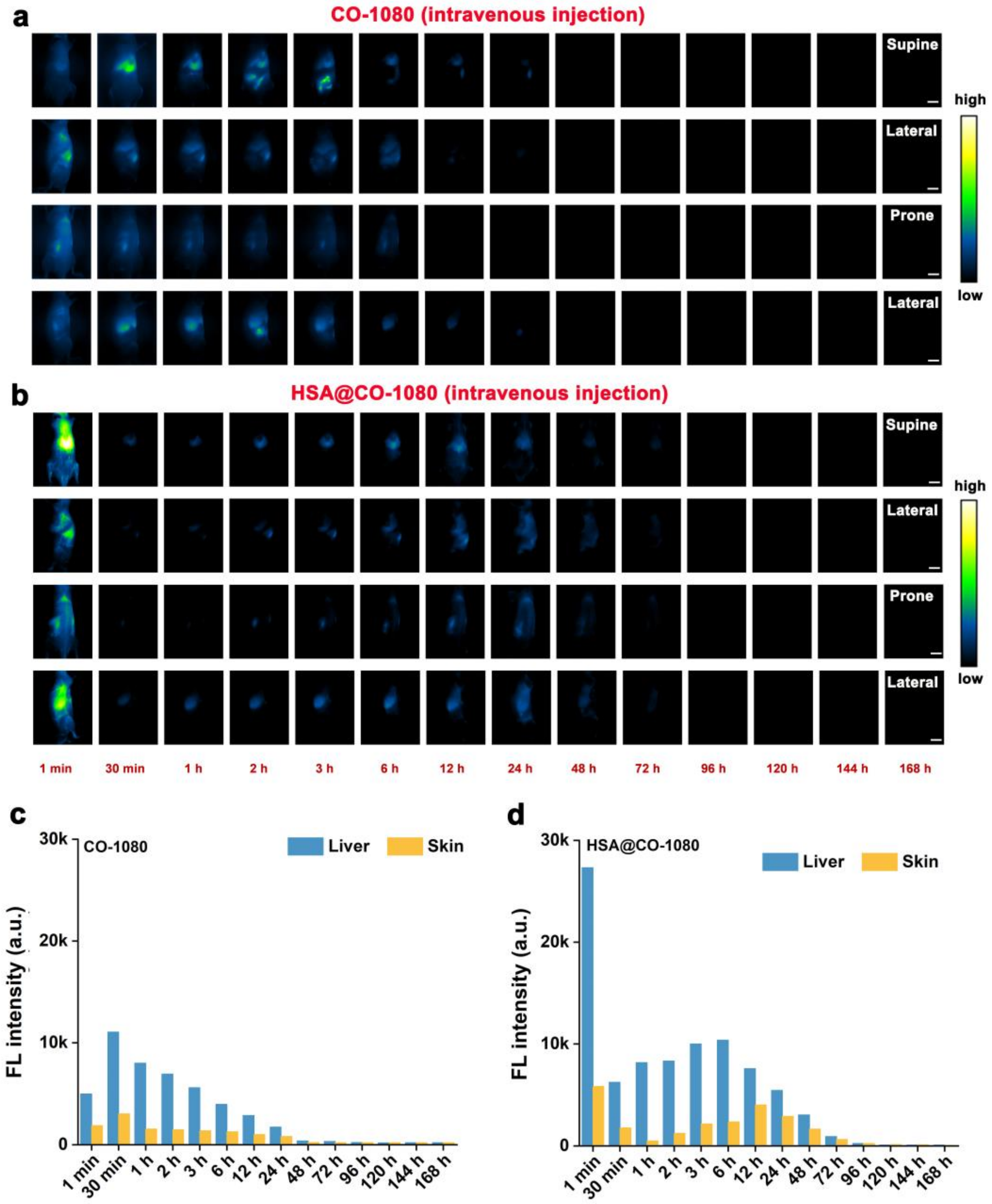

Supplementary Fig. 32. Metabolic behavior of mice injected with **a** CO-1080 and **b** HSA@CO-1080 probes through the tail vein (200 μM, 200 μL, n = 3 independent mice). Fluorescence signal statistics of mice injected with **c** CO-1080 and **d** HSA@CO-1080 probes through the tail vein. Scale bar = 1 cm. Source data are provided as a Source Data file.

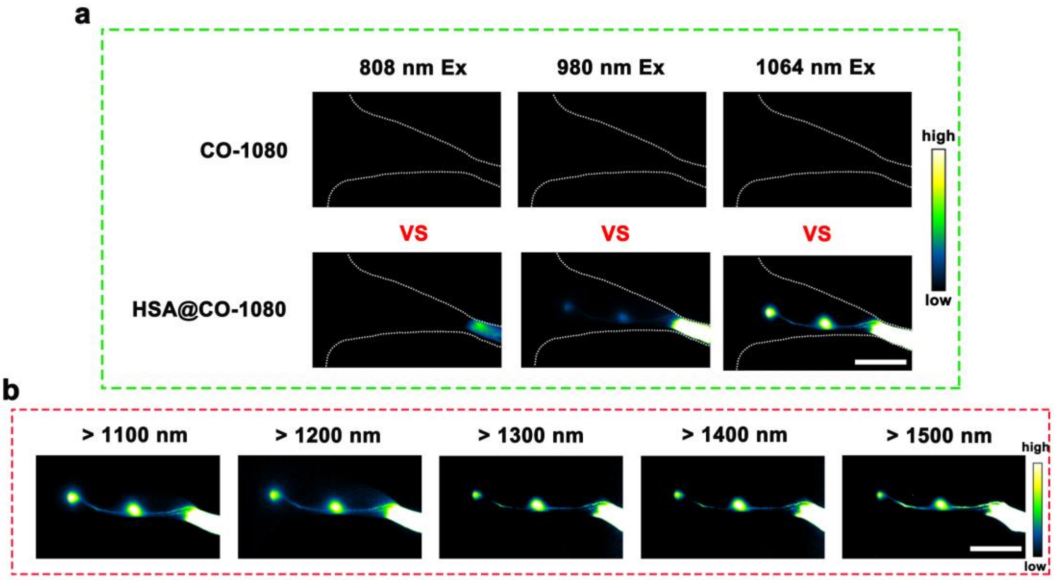

Supplementary Fig. 33. a NIR-II lymphography of the CO-1080 and HSA@CO-1080 FPs under different laser excitation (600 µM, 25 µL, n = 3 independent mice). **b** NIR-II lymphography of the CO-1080 and HSA@CO-1080 FPs under different sub-NIR-II windows (600 µM, 25 µL, n = 3 independent mice). Scale bar = 1 cm.

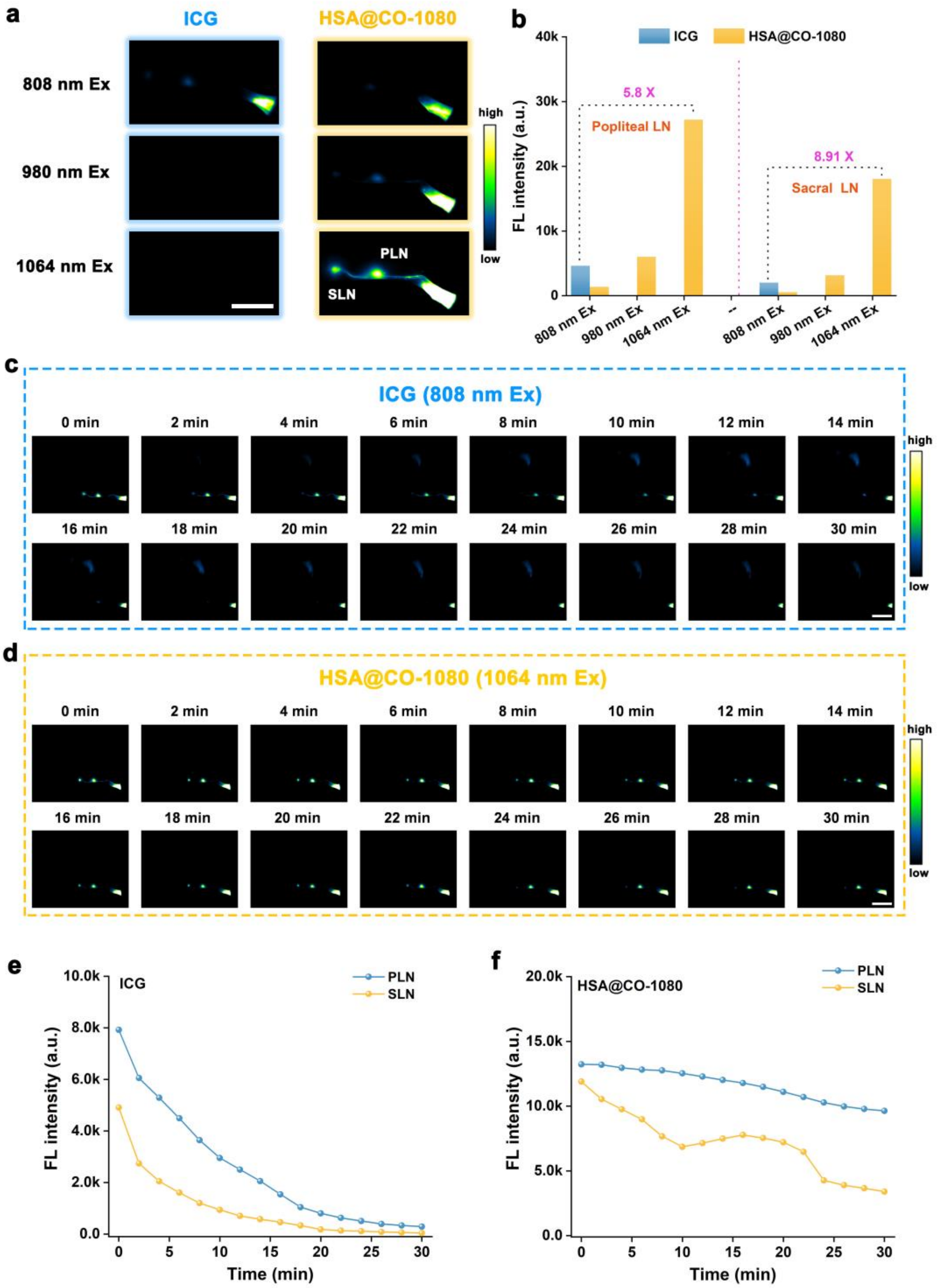

Supplementary Fig. 34. a NIR-II lymphography of the ICG and HSA@CO-1080 FPs under different laser excitations (n = 3 independent mice). **b** Comparison of NIR-II lymph node brightness between ICG and HSA@CO-1080 FPs under different laser excitations. **c-d** Images of NIR-II lymph nodes at different time points after intradermal injection of ICG and HSA@CO-1080 FPs into soles of the feet (n = 3 independent mice). **e-f** NIR-II lymph node signal statistics at different time points after intradermal injection of ICG and HSA@CO-1080 FPs into the soles of the feet (PLN: popliteal lymph node, SLN: sacral lymph node). All images were collected above 1200 nm (600 µM, 25 µL). Scale bar = 1 cm. Source data are provided as a Source Data file.

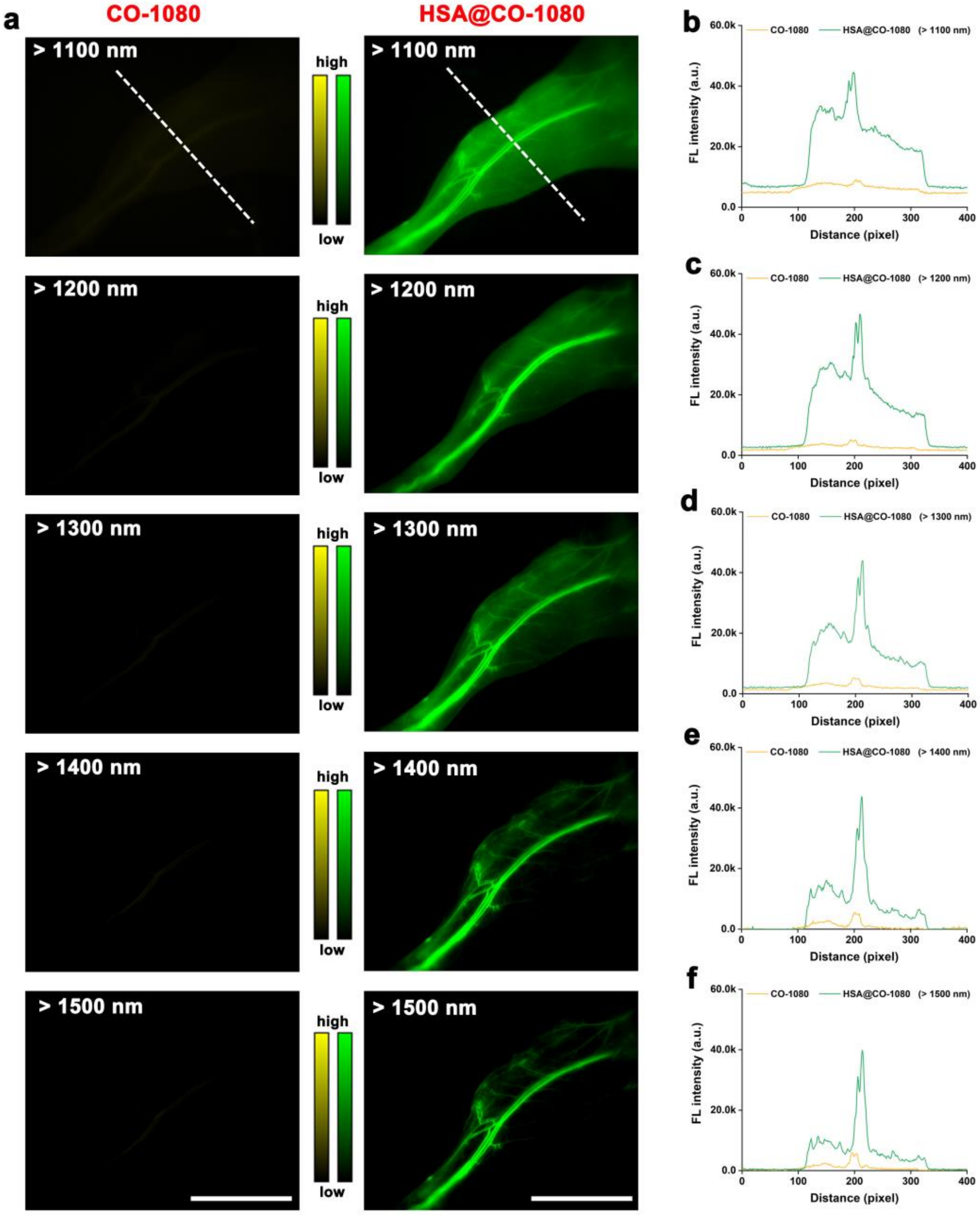

Supplementary Fig. 35. a NIR-II angiographic images of CO-1080 and HSA@CO-1080 FPs under different imaging windows (600 μM, 200 μL, n = 3 independent mice). Comparison of NIR-II angiographic signal between CO-1080 and HSA@CO-1080 FPs under **b** > 1100 nm, **c** > 1200 nm, **d** > 1300 nm, **e** > 1400 nm, and **f** > 1500 nm sub-NIR-II windows. Scale bar = 1 cm. Source data are provided as a Source Data file.

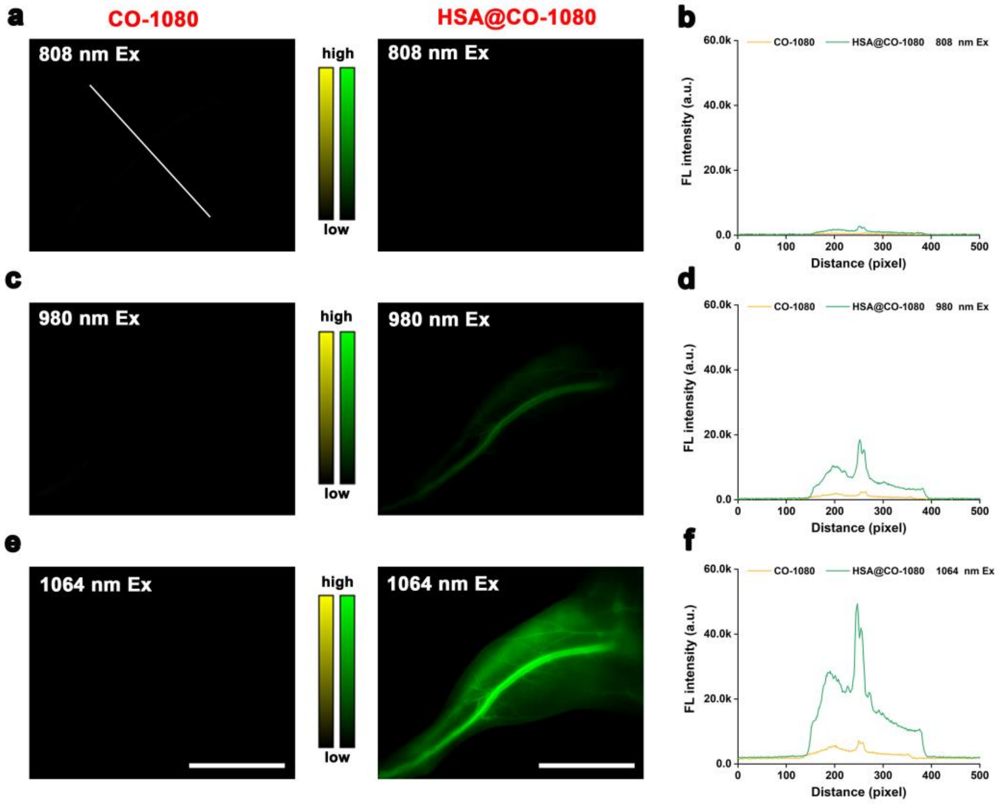

Supplementary Fig. 36. a, c, e NIR-II angiographic images of the CO-1080 and HSA@CO-1080 FPs under different laser excitation (n = 3 independent mice). **b, d, f** Comparison of NIR-II angiographic signal of the CO-1080 and HSA@CO-1080 FPs under different laser excitations. All images were collected above 1200 nm (600 μM, 200 μL). Scale bar = 1 cm. Source data are provided as a Source Data file.

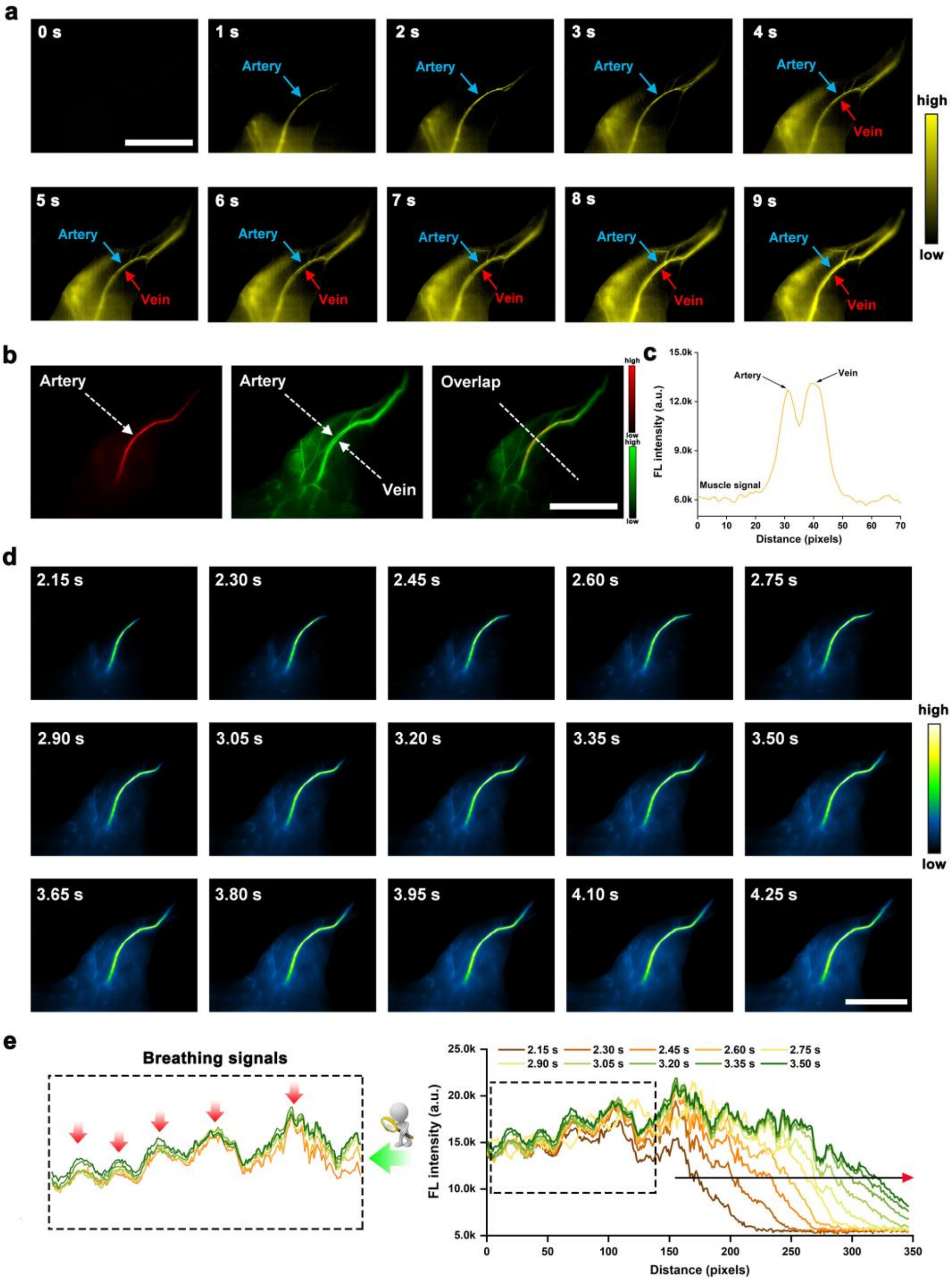

Supplementary Fig. 37. a NIR-II angiography in the short term after intravenous injection of the HSA@CO-1080 FPs. **b** Image and **c** fluorescence signal statistics of arteries and veins after intravenous injection of the HSA@CO-1080 FPs. **d** Arterial image and **e** fluorescence signal statistics in the short term after intravenous injection of the HSA@CO-1080 FPs. All images were collected above 1200 nm (600 µM, 200 µL, n = 3 independent mice). Scale bar = 1 cm. Source data are provided as a Source Data file.

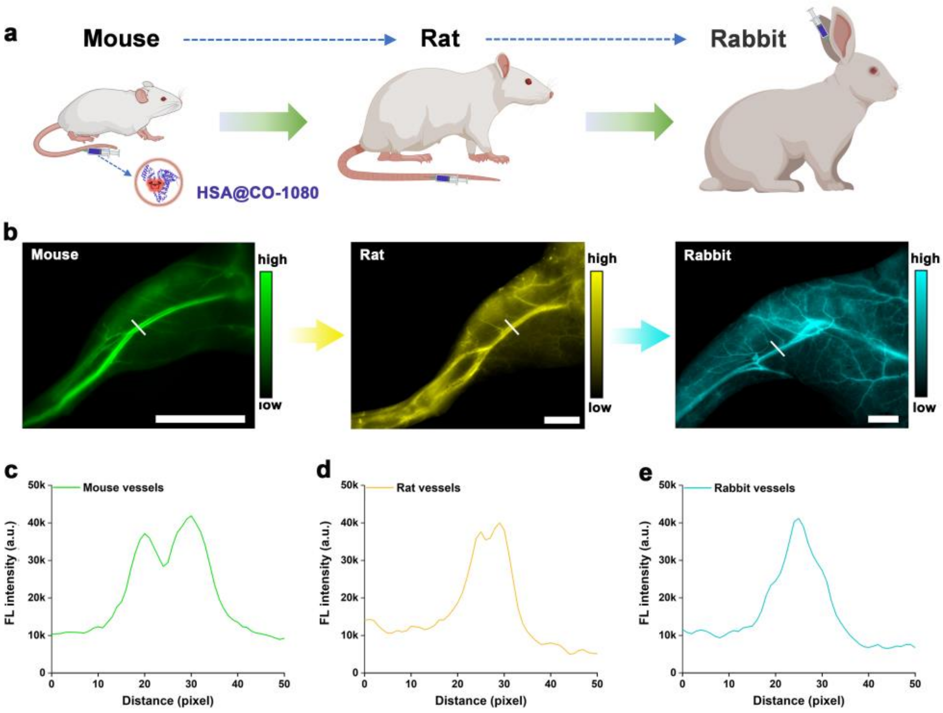

Supplementary Fig. 38. a Angiographic schematic of different animals, including mouse, rat, and rabbit. **b** NIR-II angiography images of different animals after intravenous injection of the HSA@CO-1080 FPs, including mouse, rat, and rabbit. Statistics of blood vessel signal in **c** mouse, **d** rat, and **e** rabbit after intravenous injection of the HSA@CO-1080 FPs. All images were collected above 1200 nm (600 μM, n = 3 independent experiment). Scale bar = 1 cm. Some schematic diagrams were designed using BioRender software. Source data are provided as a Source Data file.

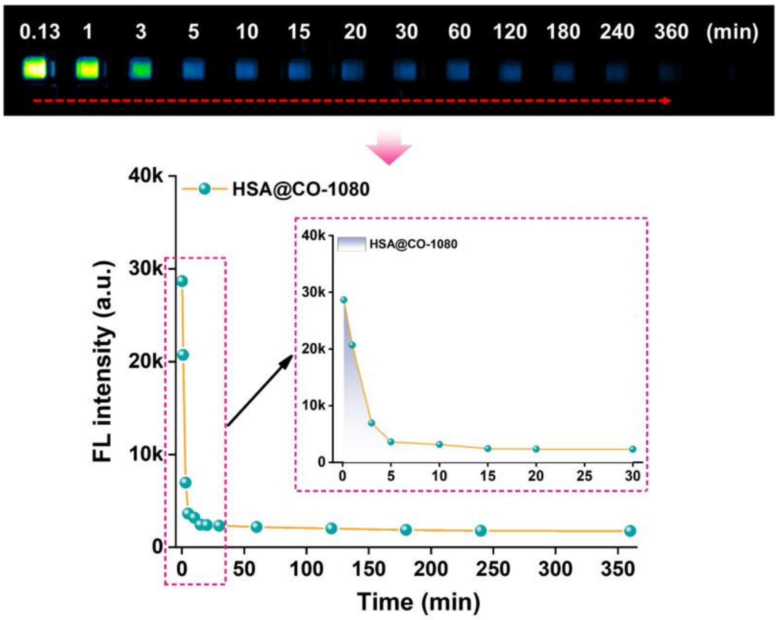

Supplementary Fig. 39. The blood half-life of HSA@CO-1080 FPs (600 μM, 200 μL, n = 3 independent mice). Source data are provided as a Source Data file.

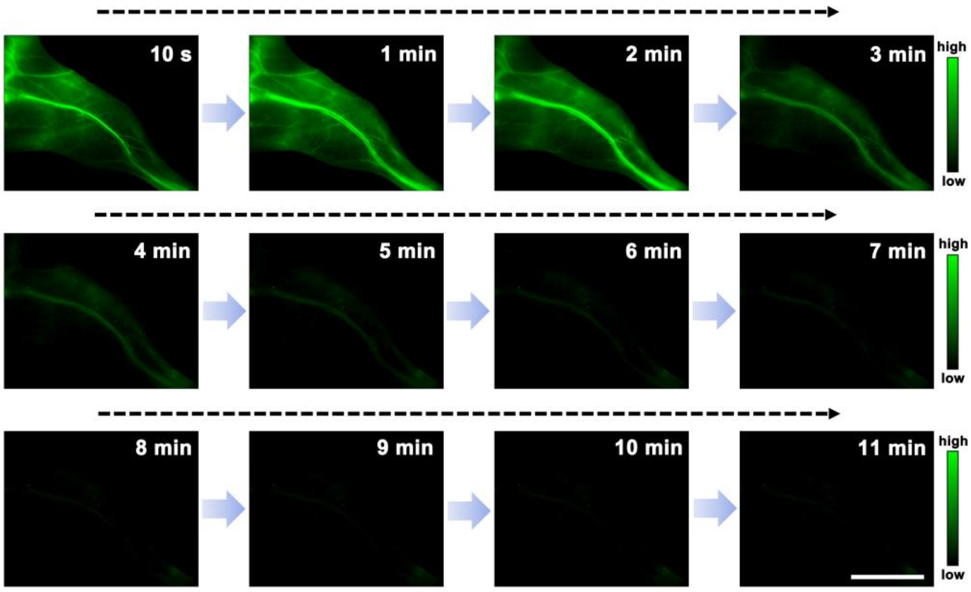

Supplementary Fig. 40. Time window for angiography of the HSA@CO-1080 FPs (600 μM, 200 μL, n = 3 independent mice). Scale bar = 1 cm.

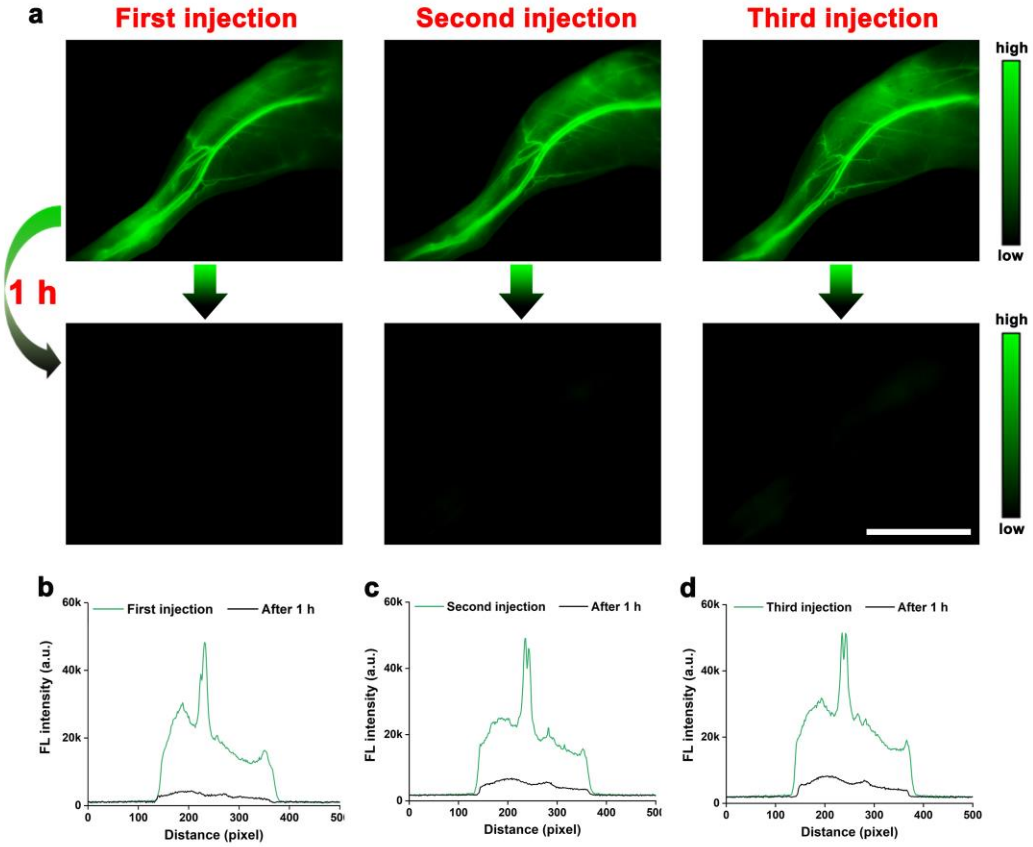

Supplementary Fig. 41. a Repeating angiography images of the HSA@CO-1080 FPs (600 μM, 200 μL, n = 3 independent mice). **b-d** Fluorescence signal statistics of repeating angiography for the HSA@CO-1080 FPs. Scale bar = 1 cm. Source data are provided as a Source Data file.

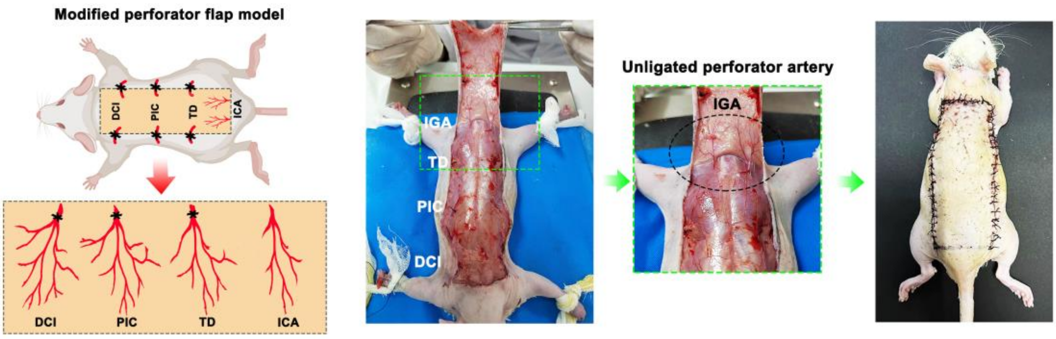

Supplementary Fig. 42. Schematic of a modified perforator flap model (DCI: deep circumflex iliac artery, PIC: posterior intercostal artery, TD: thoracodorsal artery, and IGA: inferior gluteal artery). The mouse scheme was designed using BioRender software.

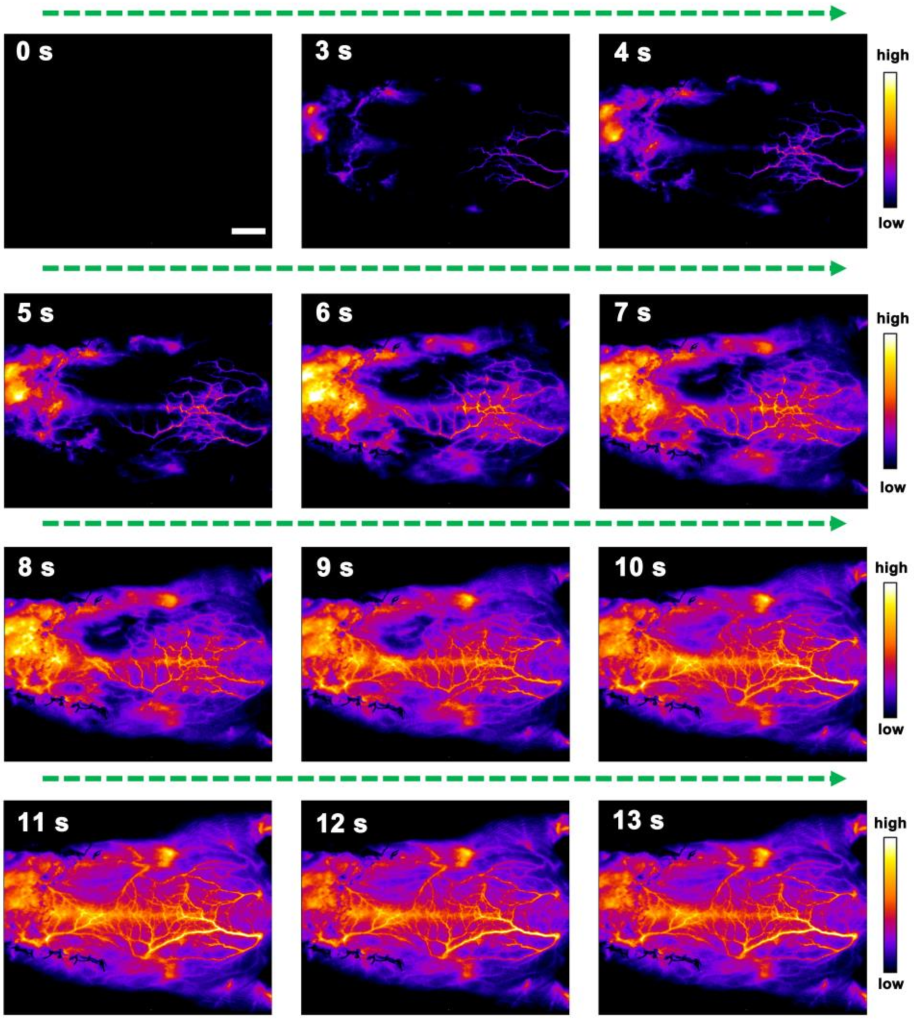

Supplementary Fig. 43. NIR-II images of the modified perforator flap at different times after intravenous injection of the HSA@CO-1080 FPs. All images were collected above 1200 nm (600 μM, 1 mL, n = 3 independent rats). Scale bar = 1 cm.

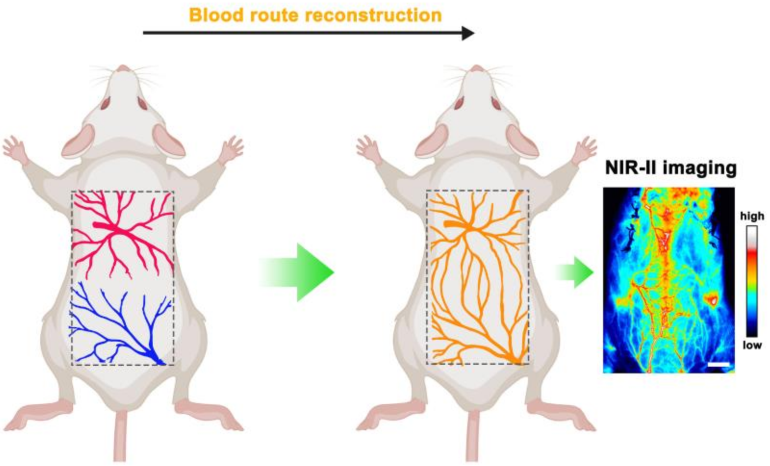

Supplementary Fig. 44. Schematic of blood route reconstruction after operation of modified perforator flap model. Scale bar = 1 cm. Schematic diagrams were designed using BioRender software.

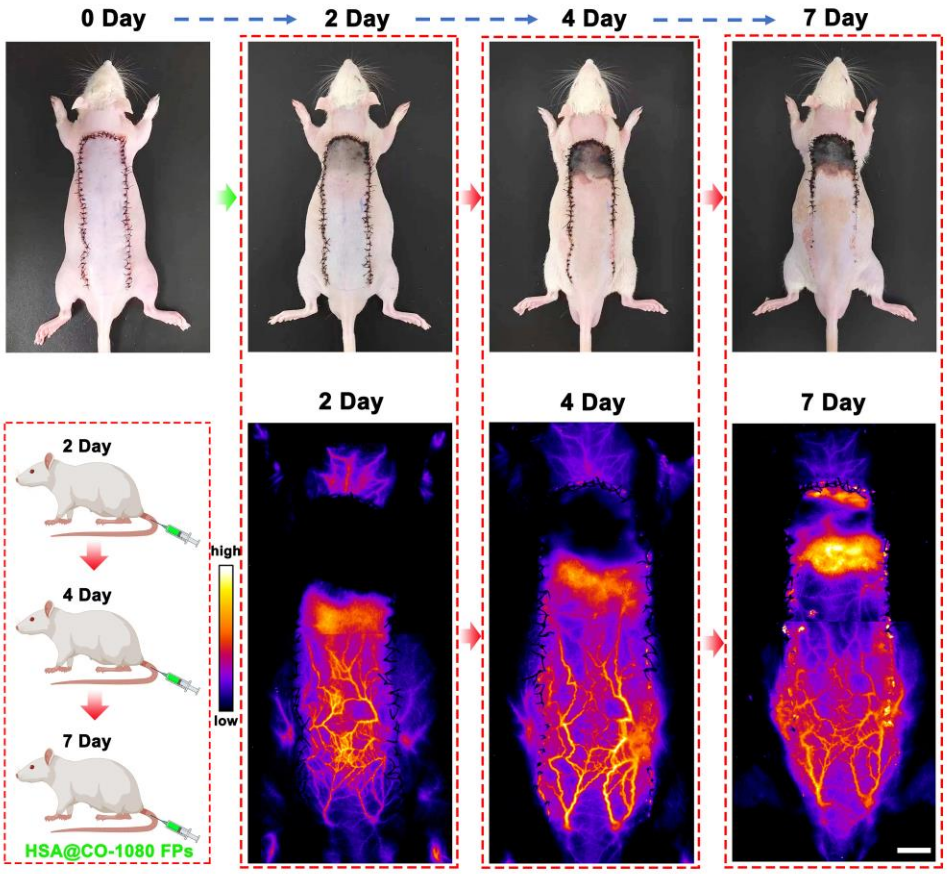

Supplementary Fig. 45. Photographs and NIR-II images of rats at different time points after the operation of the modified perforator flap model (600 µM, 1 mL, n = 3 independent rats). Scale bar = 1 cm. Schematic diagrams were designed using BioRender software.

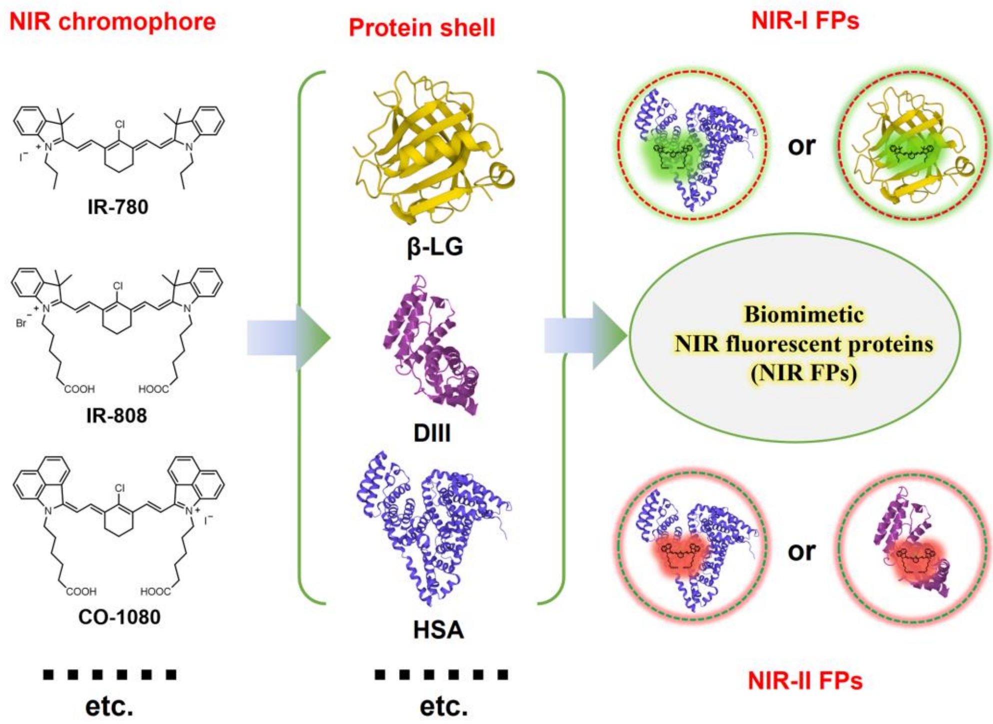

Supplementary Fig. 46. Construction schematic of biomimetic NIR-I/II fluorescent proteins (FPs). Protein structures were generated by the Protein Data Bank (PDB).

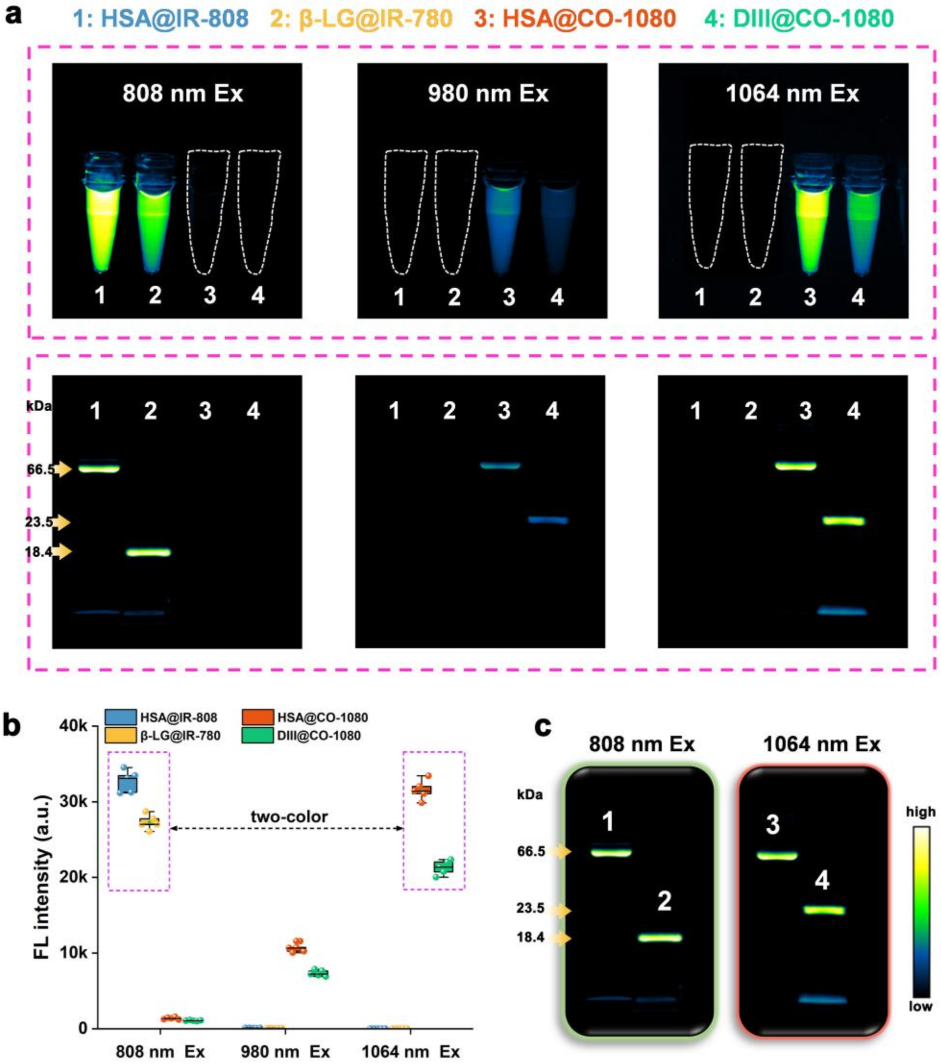

Supplementary Fig. 47. **a** Fluorescence image and SDS-PAGE gel electrophoresis of HSA@IR-808, β-LG@IR-780, HSA@CO-1080, and DIII@CO-1080 fluorescent proteins under different laser excitations (n = 4 independent experiment). **b** Fluorescence intensity of HSA@IR-808, β-LG@IR-780, HSA@CO-1080, and DIII@CO-1080 fluorescent proteins under different laser excitations (n = 5 independent samples per group). **c** SDS-PAGE gel electrophoresis of NIR-I fluorescent proteins (HSA@IR-808 and β-LG@IR-780) and NIR-II fluorescent proteins (HSA@CO-1080 and DIII@CO-1080) under 808 nm and 1064 nm laser excitation, respectively (n = 4 independent experiment). Source data are provided as a Source Data file.

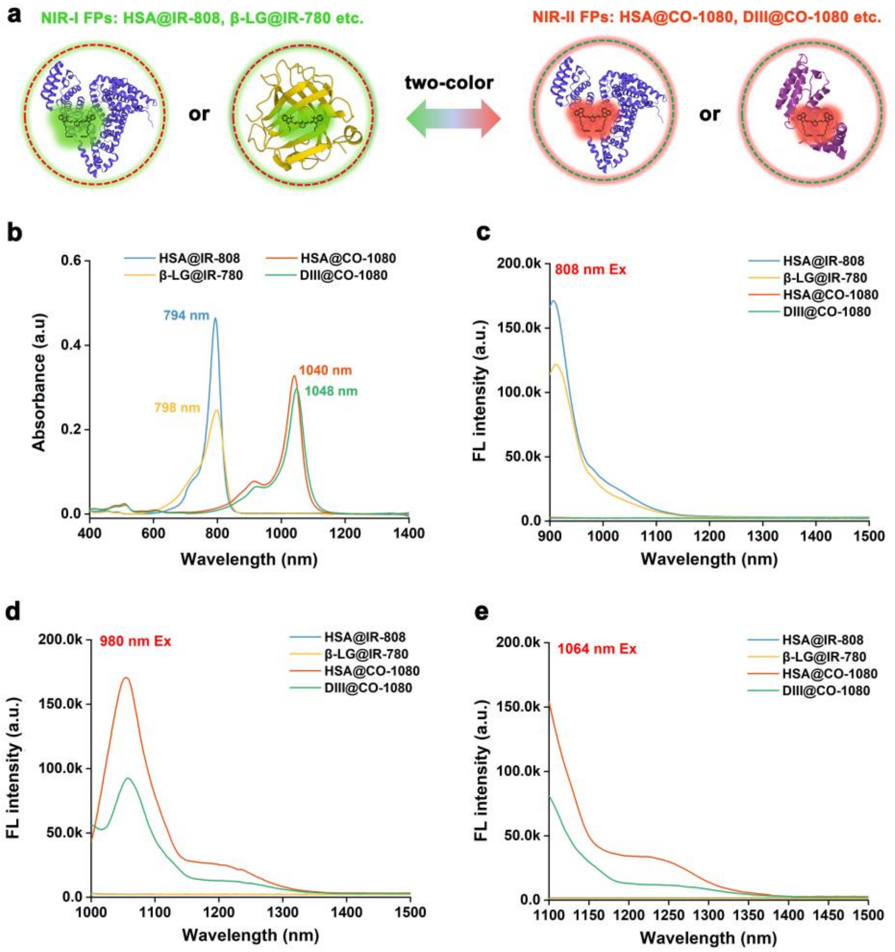

Supplementary Fig. 48. a Schematic of different NIR-I/II fluorescent protein structures, including HSA@IR-808, β-LG@IR-780, HSA@CO-1080, and DIII@CO-1080. **b** UV-absorption spectra of the HSA@IR-808, β-LG@IR-780, HSA@CO-1080, and DIII@CO-1080. Fluorescence spectra of the HSA@IR-808, β-LG@IR-780, HSA@CO-1080, and DIII@CO-1080 under **c** 808 nm, **d** 980 nm, and **e** 1064 nm laser excitations, respectively. Protein structures were generated by the Protein Data Bank (PDB). Source data are provided as a Source Data file.

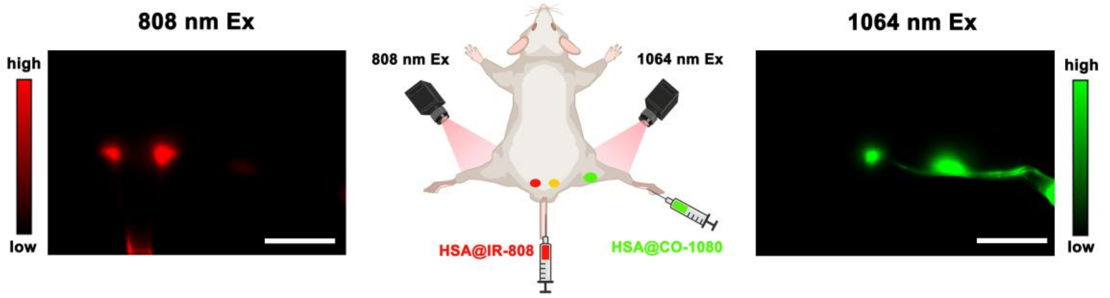

Supplementary Fig. 49. Co-localization bioimaging of lymph nodes with HSA@IR-808 FPs (intradermal tail injection) and HSA@CO-1080 FPs (intradermal footpad injection) under 808 nm and 1064 nm laser excitations, respectively. All images were collected above 1200 nm (600 µM, 25 µL, n = 3 independent mice). Scale bar = 1 cm. The mouse scheme was designed using BioRender software.

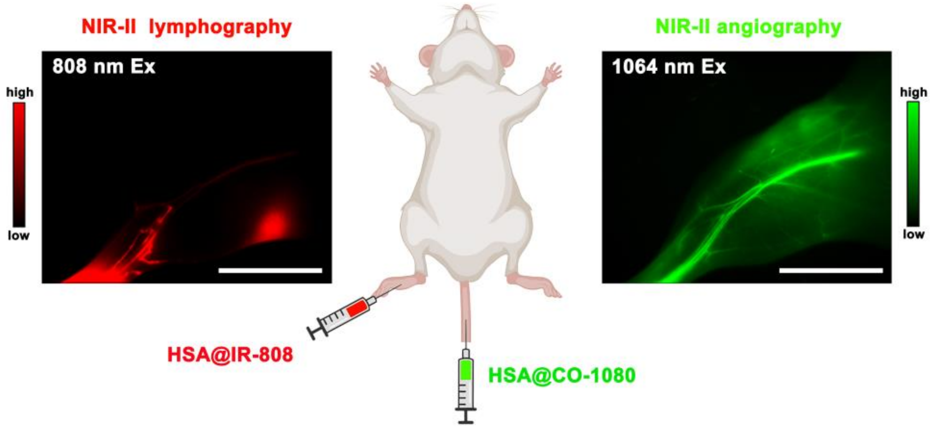

Supplementary Fig. 50. NIR-II lymphography and angiography with HSA@IR-808 FPs (intradermal footpad injection, 600 µM, 25 µL, n = 3 independent mice) and HSA@CO-1080 FPs (intravenous injection, 600 µM, 200 µL, n = 3 independent mice) under 808 nm and 1064 nm laser excitations, respectively. All images were collected above 1200 nm. Scale bar = 1 cm. The mouse scheme was designed using BioRender software.

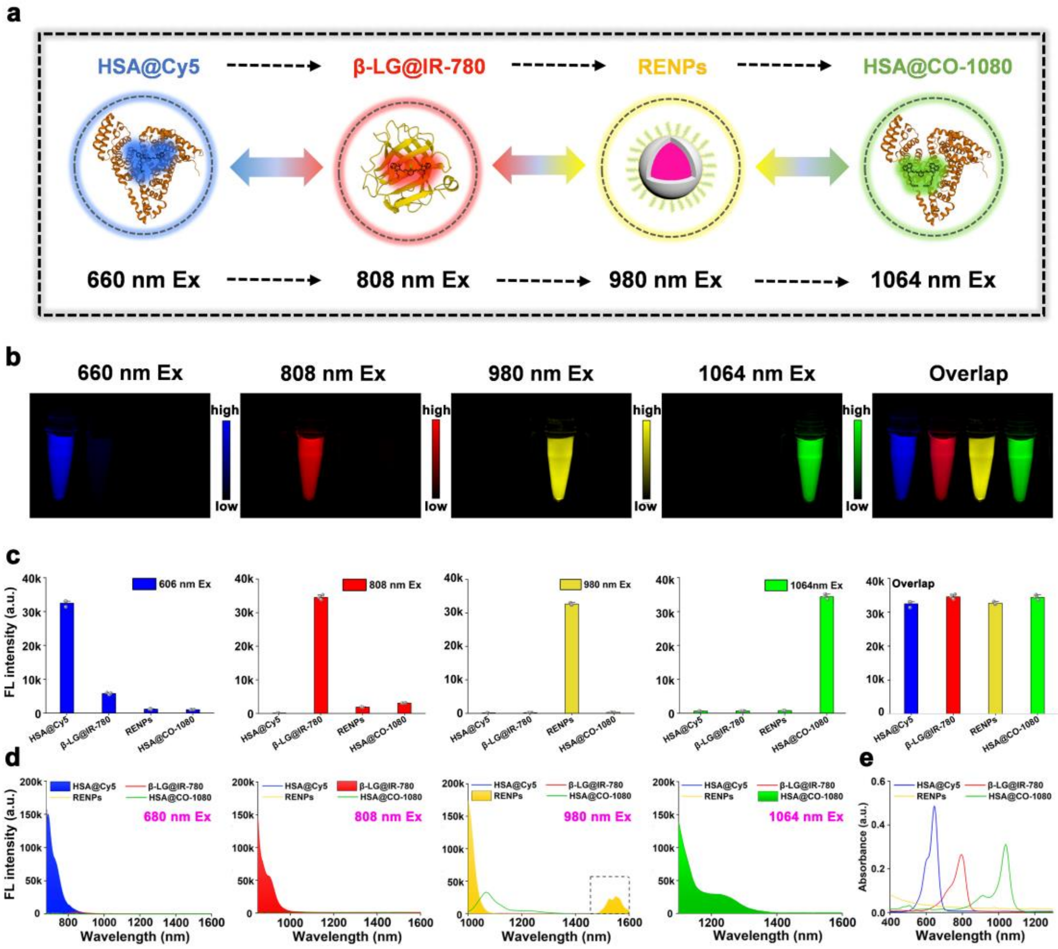

Supplementary Fig. 51. a Schematic of different NIR-I/II fluorescent probes, including HSA@Cy5, β-LG@IR-780, RENPs ($NaYbF_4$:Ce, Er@$NaYF_4$:Gd, Yb@PAA), and HSA@CO-1080. **b** Fluorescence images and **c** fluorescence intensity of the HSA@Cy5, β-LG@IR-780, RENPs ($NaYbF_4$:Ce, Er@$NaYF_4$:Gd, Yb@PAA), and HSA@CO-1080 under different laser excitations (including 660, 808, 980, and 1064 nm) (mean ± SD, n = 3 independent samples per group). **d** Fluorescence spectra of the HSA@Cy5, β-LG@IR-780, RENPs ($NaYbF_4$:Ce, Er@$NaYF_4$:Gd, Yb@PAA), and HSA@CO-1080 under different laser excitation (680, 808 nm, 980 nm, and 1064 nm). **e** UV-absorption spectra of the HSA@Cy5, β-LG@IR-780, RENPs ($NaYbF_4$:Ce, Er@$NaYF_4$:Gd, Yb@PAA), and HSA@CO-1080. Protein structures were generated by the Protein Data Bank (PDB). Source data are provided as a Source Data file.

Data Note: the imaging concentration of the RENPs probe was 250 mg/ml (> 1500 nm collection), the imaging concentration of the HSA@Cy5 probe was 10 μM (> 700 nm collection), and the imaging concentration of the β-LG@IR-780 and HSA@CO-1080 probe was 10 μM (> 1200 nm collection).
