# Supplementary material for: Biomimetic NIR-II fluorescent proteins created from chemogenic protein-seeking dyes for multicolor deep-tissue bioimaging
Source: Nat Commun. 2024 Apr 2;15:2845. doi: 10.1038/s41467-024-47063-4 (PMC10987503; doi:10.1038/s41467-024-47063-4)
Supplement: Supplementary file 2 — Reporting Summary [file 41467_2024_47063_MOESM2_ESM.pdf]

nature portfolio

Corresponding author(s): Shoujun Zhu

Last updated by author(s): Feb 23, 2024

Reporting Summary

Nature Portfolio wishes to improve the reproducibility of the work that we publish. This form provides structure for consistency and transparency in reporting. For further information on Nature Portfolio policies, see our Editorial Policies and the Editorial Policy Checklist.

Statistics

For all statistical analyses, confirm that the following items are present in the figure legend, table legend, main text, or Methods section.

n/a	Confirmed	
☐	☒	The exact sample size (n) for each experimental group/condition, given as a discrete number and unit of measurement
☐	☒	A statement on whether measurements were taken from distinct samples or whether the same sample was measured repeatedly
☐	☒	The statistical test(s) used AND whether they are one- or two-sided<br>*Only common tests should be described solely by name; describe more complex techniques in the Methods section.*
☒	☐	A description of all covariates tested
☒	☐	A description of any assumptions or corrections, such as tests of normality and adjustment for multiple comparisons
☐	☒	A full description of the statistical parameters including central tendency (e.g. means) or other basic estimates (e.g. regression coefficient) AND variation (e.g. standard deviation) or associated estimates of uncertainty (e.g. confidence intervals)
☒	☐	For null hypothesis testing, the test statistic (e.g. F, t, r) with confidence intervals, effect sizes, degrees of freedom and P value noted<br>*Give P values as exact values whenever suitable.*
☒	☐	For Bayesian analysis, information on the choice of priors and Markov chain Monte Carlo settings
☒	☐	For hierarchical and complex designs, identification of the appropriate level for tests and full reporting of outcomes
☒	☐	Estimates of effect sizes (e.g. Cohen's d, Pearson's r), indicating how they were calculated

Our web collection on statistics for biologists contains articles on many of the points above.

Software and code

Policy information about availability of computer code

Data collection	The UV-absorption spectra of different probes were measured using a LAMBDA 1050+ spectrophotometer; The NIR-II fluorescence spectra of different probes were measured by Edinburgh instrument FLS920 fluorescence spectrophotometer; Sodium dodecyl sulfate-polyacrylamide gel electrophoresis (SDS-PAGE) was performed on different probes using an American BIO-RAD electrophoresis system; The covalent binding behavior of protein to different dyes was characterized by Orbitrap Eclipse high-resolution mass spectrometer; The binding behavior of L-Cysteine to different dyes was characterized by high-resolution mass spectrometer (Agilent1290); The particle sizes of different compounds were analyzed using a Malvern Zetasizer Nano ZS size analyzer; Transmission electron microscope (TEM) images were acquired through DTM-961002 of JEOL Ltd; The 1H-NMR spectra of all dyes were obtained on Bruker AVANCE III 400 MHz NMR spectrometers; The erythrocyte absorption of hemolysis and cytotoxicity tests was detected by a microplate reader (Bio-Tek, Synergy LX, USA); NIR-I/II images were collected on a InGaAs camera (Princeton Instruments, NIRvana-640).
Data analysis	ChemDraw Professional 20.0; Graphpad prism 8; ImageJ 1.50i; Origin Pro 2018.

For manuscripts utilizing custom algorithms or software that are central to the research but not yet described in published literature, software must be made available to editors and reviewers. We strongly encourage code deposition in a community repository (e.g. GitHub). See the Nature Portfolio guidelines for submitting code & software for further information.

Data

Policy information about availability of data

All manuscripts must include a data availability statement. This statement should provide the following information, where applicable:

- Accession codes, unique identifiers, or web links for publicly available datasets
- A description of any restrictions on data availability
- For clinical datasets or third party data, please ensure that the statement adheres to our policy

All data supporting the findings of this study are available within the article and its Supplementary Information files or from the corresponding authors on reasonable request.

Research involving human participants, their data, or biological material

Policy information about studies with human participants or human data. See also policy information about sex, gender (identity/presentation), and sexual orientation and race, ethnicity and racism.

Reporting on sex and gender	N/A
Reporting on race, ethnicity, or other socially relevant groupings	N/A
Population characteristics	N/A
Recruitment	N/A
Ethics oversight	N/A

Note that full information on the approval of the study protocol must also be provided in the manuscript.

Field-specific reporting

Please select the one below that is the best fit for your research. If you are not sure, read the appropriate sections before making your selection.

☒ Life sciences ☐ Behavioural & social sciences ☐ Ecological, evolutionary & environmental sciences

For a reference copy of the document with all sections, see nature.com/documents/nr-reporting-summary-flat.pdf

Life sciences study design

All studies must disclose on these points even when the disclosure is negative.

Sample size	Sample sizes were not chosen based on statistical method. Samples sized was chosen as large as possible to be sufficient to obtain statistics (n= 3-15). For in vitro experiments, samples were prepared and measured at least thrice. For in vivo experiments, each group has at least 3 biologically independent mice. All data are reported as mean ± standard deviation (SD) from at least three independent runs. Statistical significance was determined by a two-tailed Student's t-test, with a p-value of less than 0.05 considered significant. Fig.1b n=5, Fig.1c n=5, Fig.1j n=4, Fig.1k n=5, Fig.1m n=3, Fig.2b n=5, Fig.2d,e n=15, Fig.2k n=4, Fig.3b n=10, Fig.3c n=5, Fig.3f n=4, Fig.5b n=3, Fig.5c n=3, Fig.5d n=3, Fig.5g n=3, Fig.6c n=4, Fig.6d n=10, Fig.6e n=3, Fig.6f n=3, Fig.6g n=3, Fig.6h n=3, Fig.6j n=3, Fig.S6a,d,g n=10, Fig.S6b,e,g,h n=4, Fig.S7b,d,e n=10, Fig.S7g,h n=4, Fig.S11a-c n=3, Fig.S11a-c n=3, Fig.S11e n=5, Fig.S31a-c n=3, Fig.S31d-i n=4, Fig.S32a,b n=3, Fig.S33a,b n=3, Fig.S34a,c,d n=3, Fig.S35a n=3, Fig.S36a,c,e n=3, Fig.S37a,b,d n=3, Fig.S38b n=3, Fig.S39 n=3, Fig.S40 n=3, Fig.S41 n=3, Fig.S43 n=3, Fig.S45 n=3, Fig.S47a,c n=4, Fig.S47b n=5, Fig.S49 n=3, Fig.S50 n=3, Fig.S51b n=3.
Data exclusions	No data was excluded.
Replication	Every experiment at replication was successful. All experiments were performed a minimum of three replicates in independent experiments with similar results.
Randomization	Randomly grouped
Blinding	The investigators were blinded to group allocation during data collection and analysis.

Reporting for specific materials, systems and methods

We require information from authors about some types of materials, experimental systems and methods used in many studies. Here, indicate whether each material, system or method listed is relevant to your study. If you are not sure if a list item applies to your research, read the appropriate section before selecting a response.

Materials & experimental systems

n/a	Involved in the study
☒	☐ Antibodies
☐	☒ Eukaryotic cell lines
☒	☐ Palaeontology and archaeology
☐	☒ Animals and other organisms
☒	☐ Clinical data
☒	☐ Dual use research of concern
☐	☐ Plants

Methods

n/a	Involved in the study
☒	☐ ChIP-seq
☒	☐ Flow cytometry
☒	☐ MRI-based neuroimaging

Eukaryotic cell lines

Policy information about cell lines and Sex and Gender in Research

Cell line source(s)	Mouse breast cancer cell line (4T1) was obtained from the Shanghai Enzyme Research Biotechnology Co., LTD (Shanghai, China); Mouse fibroblasts cell line (L929) was kindly provided by Joint Laboratory of Opto-Functional Theranostics in Medicine and Chemistry, First Hospital of Jilin University.
Authentication	None of the cell lines used were authenticated.
Mycoplasma contamination	Negative for mycoplasma
Commonly misidentified lines (See ICLAC register)	No misidentified lines

Animals and other research organisms

Policy information about studies involving animals; ARRIVE guidelines recommended for reporting animal research, and Sex and Gender in Research

Laboratory animals	Balb/c (female, 6-8 weeks), and C57 mice (female, 6-8 weeks), SD rats (male, 250-300 g), and Japanese big-ear rabbits (female, 1.5-2 Kg) were purchased from Liaoning Changsheng Biotechnology Co., Ltd and Beijing Vital River Laboratory Animal Technology Co., Ltd. Bedding, nesting material, food, and water were provided ad libitum, and changed and replenished as required. The feeding environment was 20-22°C, 35-45% humidity, 12 h light-dark alternation.
Wild animals	No wild animals were used in this study.
Reporting on sex	Balb/c and C57 mice, and Japanese big-ear rabbits were female gender; SD rats were male gender.
Field-collected samples	No filed-collection
Ethics oversight	All animal experiments were conducted according to the protocols approved by the Institutional Animal Care and Utilization Committee of Jilin University (Procedure Number: 20210642).

Note that full information on the approval of the study protocol must also be provided in the manuscript.

Plants

Seed stocks	N/A
Novel plant genotypes	N/A
Authentication	N/A
