# Supplementary material for: Biomimetic NIR-II fluorescent proteins created from chemogenic protein-seeking dyes for multicolor deep-tissue bioimaging
Source: Nat Commun. 2024 Apr 2;15:2845. doi: 10.1038/s41467-024-47063-4 (PMC10987503; doi:10.1038/s41467-024-47063-4)
Supplement: Supplementary file 4 — Description of Additional Supplementary Files [file 41467_2024_47063_MOESM4_ESM.pdf]

Supplementary Movie 1:
Molecular dynamics simulation (MD) optimization for the screened covalent-bond conformation of HSA@CO-1080 using Desmond module.
